# A High-Quality Haplotype-Resolved Genome of Common Bermudagrass (*Cynodon dactylon* L.) Provides Insights Into Polyploid Genome Stability and Prostrate Growth

**DOI:** 10.3389/fpls.2022.890980

**Published:** 2022-04-25

**Authors:** Bing Zhang, Si Chen, Jianxiu Liu, Yong-Bin Yan, Jingbo Chen, Dandan Li, Jin-Yuan Liu

**Affiliations:** ^1^School of Life Sciences, Tsinghua University, Beijing, China; ^2^College of Animal Science and Technology, Yangzhou University, Yangzhou, China; ^3^Institute of Botany, Jiangsu Province and Chinese Academy of Sciences, Nanjing, China

**Keywords:** *Cynodon dactylon*, common bermudagrass, genome, haplotype, tiller angle

## Abstract

Common bermudagrass (*Cynodon dactylon* L.) is an important perennial warm-season turfgrass species with great economic value. However, the reference genome is still deficient in *C. dactylon*, which severely impedes basic studies and breeding studies. In this study, a high-quality haplotype-resolved genome of *C. dactylon* cultivar Yangjiang was successfully assembled using a combination of multiple sequencing strategies. The assembled genome is approximately 1.01 Gb in size and is comprised of 36 pseudo chromosomes belonging to four haplotypes. In total, 76,879 protein-coding genes and 529,092 repeat sequences were annotated in the assembled genome. Evolution analysis indicated that *C. dactylon* underwent two rounds of whole-genome duplication events, whereas syntenic and transcriptome analysis revealed that global subgenome dominance was absent among the four haplotypes. Genome-wide gene family analyses further indicated that homologous recombination-regulating genes and tiller-angle-regulating genes all showed an adaptive evolution in *C. dactylon*, providing insights into genome-scale regulation of polyploid genome stability and prostrate growth. These results not only facilitate a better understanding of the complex genome composition and unique plant architectural characteristics of common bermudagrass, but also offer a valuable resource for comparative genome analyses of turfgrasses and other plant species.

## Introduction

Common bermudagrass (*Cynodon dactylon* L., 2n = 4x = 36) is an important warm-season turfgrass species and is widely used to produce beautiful and uniform turf for public parks, home lawns, golf courses, and sport fields in warm regions around the world (Yang et al., 2018; Zhang et al., 2018a). In some regions, *C. dactylon* is also used as forage, medicinal, and energy plants (Hill et al., 2001; Nagori and Solanki, 2011; Xu et al., 2011). Since its origination from Africa or Indo-Malaysian, *C. dactylon* was spread to tropical and subtropical areas worldwidely (Kneebone, 1966; Harlan and de Wet, 1969). As a cross-pollinating plant, wild germplasms of *C. dactylon* collected at different regions usually show enormous genetical and morphological variations (Wu et al., 2004, 2007; Farsani et al., 2012; Tan et al., 2014; Zheng et al., 2017). Karyotype and molecular marker analyses not only revealed that polyploidy and aneuploidy events exist in *C. dactylon* but also pointed out the genome of *C. dactylon* is highly heterozygous (Wu et al., 2006; Chaves et al., 2019; Grossman et al., 2021). These characteristics make *C. dactylon* an interesting plant material to investigate genome stability, variability, and evolution (Khanal et al., 2017).

Unlike domesticated cereal grasses including rice, wheat, maize, and sorghum, *C. dactylon* has typical plant architectural characteristics of wild grasses that its stems are differentiated into shoots, stolons, and rhizomes (Dong and de Kroon, 1994; Zhang et al., 2019; Ma et al., 2021). Shoots grow erectly and are widely seen in other plants, whereas stolons and rhizomes are two types of prostrate stems that grow aboveground and underground, respectively (Guo et al., 2021). Through regeneration of new seedlings at stolon nodes, *C. dactylon* plants are asexually reproduced in a colonial growth mode (Zhang and Liu, 2018). The high efficiency to build turf using commercial *C. dactylon* cultivars is mainly derived from this virtue. During cold days in winter, the aboveground parts of common bermudagrass plants withered and died, whereas the underground rhizomes remain alive and new plants will regenerate from rhizome nodes at warm days next year (Satorre et al., 1996). By repeating the cycle of growth at aboveground and dormancy at underground, *C. dactylon* maintains a perennial life style, which also contribute to its usage as an eminent turfgrass. Development of asexual reproductive and perennial versions of important grain crops is an attractive measure to sustainably meet the increasing global food demand (Glover et al., 2010; Ozias-Akins and Conner, 2020). Elucidating the mechanism how *C. dactylon* possesses its unique plant architectural characteristics could simultaneously provide new insights into turf breeding and crop improvement.

In this study, we reported a haplotype-resolved assembly of the highly heterozygous *C. dactylon* genome through the combined application of Pacific Biosciences (PacBio) single-molecule sequencing, Illumina paired-end sequencing, Bionano optical mapping, and chromosome conformation capture (Hi-C) technologies. With the assembled genome dataset and annotation information, we further analyzed the subgenomic composition and adaptive evolution of *C. dactylon*. Results of this study not only expand our understanding of genome structure and plant architectural regulation in *C. dactylon*, but also provide a valuable resource for genetic studies and breeding of turfgrasses.

## Materials and Methods

### Plant Materials and Growth Conditions

*Cynodon dactylon* cultivar Yangjiang was used for genome sequencing and assembly in this study. The bermudagrass turf were grown in turfgrass plots of Yangzhou University (longitude and latitude: 32°35’N, 119°40′E; average annual temperatures: 22.4°C; average annual precipitation: 1, 106 mm; annual average sunshine hours: 1, 960 h; soil type: 80% river sand; and 20% peat soil) under routine management conditions (irrigation: keep the soil moist as required; fertilization: four times/year; and mowing: one times/month) for 3 years. Healthy leaves were randomly collected from the turf plots. Half of the leaf samples were frozen and used for *de novo* sequencing, whereas another half of fresh leaf samples were used for Bionano and Hi-C sequencing. *Oryza sativa* subspecies *indica* cultivar 93–11 was grown in growth chamber at 24°C under 16 h/8 h light/dark conditions.

### Flow Cytometry Estimation of Genome Size

The genome size of *C. dactylon* cultivar Yangjiang was estimated using flow cytometry as previously described (Zhang et al., 2020). Specifically, *O*. *sativa* cv. 93–11 with a genome size of 430 Mb was used as an internal standard. Young leaves of *C. dactylon* and *O. sativa* were homogenized on ice in Galbraith’s buffer (45 mM MgCl_2_, 30 mM sodium citrate, 20 mM MOPS, and 0.1% (v/v) Triton X-100, pH 7.0) with 50 μg mL^−1^ propidium iodide. After filtration with 40 μm nylon cell strainer (BD Biosciences, Franklin Lakes, United States), samples were analyzed on a FACSCanto^™^ II flow cytometer (BD Biosciences). The flow cytometry data were analyzed using BD Spectrum Viewer.

### Illumina Sequencing and K-mer Analysis

Genomic DNA was isolated from the frozen leave samples using the DNeasy Plant Mini Kit (Qiagen, Hilden, Germany). The DNA quality and concentration were tested by 1% agarose gel electrophoresis and Qubit 2.0 Fluorometer (Life Technologies, Carlsbad, United States). Two paired-end libraries with short insert size of 270 bp and 500 bp were constructed using the NEBNext^®^ Ultra^™^ DNA Library Prep Kit for Illumina^®^ (New England Biolabs, Ipswich, United States) and sequenced on the Illumina HiSeq X Ten platform (Illumina, San Diego, United States). The raw Illumina sequencing reads were processed with SOAPnuke v2.1.6^1^ to remove adapters and low-quality reads (Chen et al., 2018). The obtained 161.1 Gb high-quality sequencing reads were used to generate a k-mer depth distribution curve adopting the Jellyfish v2.3.0.^2^ The obtained peak k-mer number (*k* = 27) and corresponding peak depth were calculated by GenomeScope v2.0^3^ to estimate the genome size and heterozygosity (Marçais and Kingsford, 2011).

### PacBio Sequencing and Preliminary Genome Assembly

High-molecular weight (HMW) DNA fragments were separated from the extracted genomic DNA samples using BluePippin Size Selection System (Sage Science, Beverly, United States) through pulse-field gel electrophoresis and eight 20-kb sequencing libraries were constructed using SMRTbell Template Prep Kit (Pacific Biosciences, Menlo Park, United States) following the manufacturer’s instructions. The libraries (16 SMRT cells) were sequenced on the PacBio RSII platform (Pacific Biosciences). Contig sequences were assembled from the 151.99 Gb PacBio sequencing reads using Hifiasm v0.12^4^ and polished by Racon v1.4.3^5^ (Cheng et al., 2021). The Illumina sequencing reads were aligned to the assembled contigs using Bwa-mem v2.2.1^6^ and the draft assembly was corrected by the aligned short sequences using Pilon v1.24^7^ (Walker et al., 2014).

### Bionano Optical Genome Mapping

HMW DNA was extracted from the agarose-embedded cell nuclei fractions, which were isolated from fresh leaf samples, using the Bionano Prep^™^ Plant DNA Isolation Kit (Bionano Genomics, San Diego, United States) following the manufacturer’s instructions. The DNA was digested by the single-stranded nicking endonuclease Nt.BspQI, fluorescently labeled, loaded into a Saphyr Chip^®^, and imaged on a Saphyr Optical Genome Mapping Instrument (Bionano Genomics). The 395.4 Gb image data were filtered using a molecule length cutoff of 100 kb and a label number cutoff of 6, and assembled to 954 genome maps. To assist genome assembly, contigs obtained from the above-mentioned PacBio sequencing were transformed into *in silico Bsp*QI-digested reference genome maps and compared with the optical genome maps. The aligned and merged genome maps were further transformed into scaffold sequences using the Bionano Solve^™^ v3.6.1.^8^

### Hi-C Sequencing and Pseudochromosome Construction

Fresh leaf samples were fixed in 1% formaldehyde to maintain the 3-D structure of genome. Genomic DNA was extracted and digested with restriction endonuclease MboI. The digested DNA fragments were biotin-labeled at the ends and ligated to each other randomly. The ligated DNA was sheared into 300–600 bp fragments, blunt-end repaired, and purified using streptavidin pull-down. The purified DNA was also sequenced on the Illumina HiSeq X Ten platform, which yielded 231.38 Gb of data with 771 million paired-end reads. The paired-end reads were mapped to the assembled scaffold sequences using Juicer v1.6^9^ to discriminate valid and invalid interaction pairs (Durand et al., 2016). The obtained 185 million valid interaction pairs (55.5 Gb data) were further used to adjust the relative locations of the scaffolds and cluster the scaffolds into pseudochromosomes using 3D-DNA^10^ (Kronenberg et al., 2021).

### Annotation and Analysis of Repetitive Sequences

Repetitive sequences were annotated by combining the homology alignment and *de novo* prediction approaches (Zhang et al., 2021). For the homology alignment approach, the assembled genome sequence was blast searched against the RepBase repeat sequence collection^11^ using RepeatMasker v4.0.9^12^ (Tempel, 2012). For the *de novo* prediction approach, five softwares, including RepeatModeler,^13^ PILER,^14^ RepeatScout,^15^ LTR_Finder,^16^ and Tandem Repeats Finder,^17^ were used to find the possible repeat sequences (Price et al., 2005; Flynn et al., 2020). The identified repetitive sequences were manually checked and classified according to the nomenclature system of transposons. The insertion time of different families of long-terminal repeat retrotransposons (LTR-RTs) were calculated using the formula *T* = *k*/2*r*, where *k* is the divergence distance between the 5′ LTR and 3′ LTR of intact LTR-RTs and *r* is the base substitution rate (1.38 × 10^−8^ substitutions/site/year for grasses; Ma et al., 2004). The LTR Assembly Index (LAI) scores of assembled pseudo chromosomes and whole genome were calculated using LTR_retriever v2.9.0^18^ with default parameters (Ou et al., 2018). Putative centromeric repeat arrays were specifically identified using Tandem Repeats Finder with searching parameters “1 1 2 80 5 200 2000 -d –h” as previously described (VanBuren et al., 2020). The identified centromeric repeat array sequences were used to construct a maximum likelihood phylogenetic tree using MEGA v10.0.5 with a bootstrap of 1,000.

### Prediction and Annotation of Protein-Coding Genes

Protein-coding genes were identified by combining the homology alignment prediction, *ab initio* prediction, and transcriptome-assisted prediction approaches (Zhang et al., 2021). For the homology alignment approach, protein sequences of *Arabidopsis thaliana* and five grass species, including *O. sativa*, *Brachypodium distachyon*, *Zea mays*, *Sorghum bicolor*, and *Oropetium thomaeum*, were downloaded from the Phytozome database^19^ and blast searched against the assembled genome sequence to identify the homologous proteins, which were then aligned to the genome by GeneWise^20^ to annotate gene structures (Birney et al., 2004). *Ab initio* gene prediction was conducted using five softwares, including Augustus v3.4.0,^21^ geneid v1.4.4,^22^ FgeneSH,^23^ GlimmerHMM v3.0.4,^24^ and Genscan^25^ with default parameters (Yao et al., 2005; Nachtweide and Stanke, 2019). For transcriptome-assisted prediction, the PacBio single-molecule transcriptome sequencing data of mixed organ samples (Zhang et al., 2018a) were aligned to the assembled genome using GMAP^26^ and the gene structures were modeled using PASA,^27^ whereas Illumina transcriptome sequencing data of six different organs (Chen et al., 2021) were aligned to the genome using TopHat v2.1.1^28^ and the gene structures were modeled using Cufflinks v2.2.1^29^ (Wu and Watanabe, 2005; Ghosh and Chan, 2016). A non-redundant reference gene set was generated by merging the predicted genes using EVidenceModeler v1.1.1^30^ (Haas et al., 2008). Functional annotations of the reference gene set were obtained through orthology assignment of the eggNOG v5.0 database^31^ using eggNOG-mapper v2^32^ (Cantalapiedra et al., 2021). Gene Ontology (GO) and Kyoto Encyclopedia of Genes and Genomes (KEGG) annotation of the reference gene set were obtained through BLAST searching against the GO database^33^ and the KEGG pathway database,^34^ respectively. KEGG enrichment analysis were performed using KEGG-Orthology Based Annotation System (KOBAS; Xie et al., 2011).^35^ Transcription factors (TFs) were annotated using iTAK^36^ incorporated with PlantTFDB database^37^ (Zheng et al., 2016).

### Prediction of Non-coding RNA Genes

rRNA and tRNA genes were predicted using the programs Barrnap^38^ and tRNAscan-SE-2.0,^39^ respectively (Chan et al., 2021). miRNA, snoRNA, and snRNA genes were all identified by searching against the Rfam database *via* Infernal v1.1.4^40^ with default parameters (Nawrocki and Eddy, 2013).

### BUSCO Assessment

The completeness and accuracy of the assembled genome and predicted reference gene set were both assessed using the embryophyta_odb10 core gene collect (1,375 genes) of the Benchmarking Universal Single-Copy Orthologs (BUSCO) v5.2.2 database^41^ (Simão et al., 2015). The number of single-copy and duplicated genes with complete coverage, genes with fragment coverage, and missing genes were all counted.

### Gene Family Identification, Phylogenetic Analysis, and Divergence Time Estimation

The protein sequences of *A. thaliana*, *O. sativa*, *B. distachyon*, *Z. mays*, *S. bicolor*, *O. thomaeum*, *Panicum hallii*, *Setaria viridis*, *Hordeum vulgare*, and *Tritcum urartu* were downloaded from the Phytozome database. Orthologous gene families were clustered using OrthoFinder v2.5.4^42^ through all-against-all blast alignment of these protein sequences and predicted protein sequences of *C. dactylon* (Emms and Kelly, 2015). The identified 112 single-copy orthologous gene families were aligned using MUSCLE^43^ and the alignments of each gene family were concatenated to a super-alignment matrix. A phylogenetic tree was then constructed using OrthoFinder with *A. thaliana* as the outgroup. PAML v4.9^44^ was used to estimate the divergence time of *C. dactylon* using recorded divergence times of other 10 species in the TimeTree database^45^ as calibrations (Yang, 2007).

### Synteny and WGD Analysis

Homologous pairs of *C. dactylon* proteins were identified using the all-to-all search in BLASTP v2.12.0^46^ with an E-value cutoff of 10^−5^. Syntenic blocks with at least 50 collinear gene pairs were then identified using MCScanX^47^ with default parameters (Wang et al., 2012). The same method was used to identify the collinear blocks between *C. dactylon* and *O. thomaeum*/*B. distachyon*. Synonymous substitutions per site values (Ks) of syntenic gene pairs were calculated using PAML v4.9 and the distribution of Ks values was plotted to infer the time for speciation or whole-genome duplication (WGD) events using the formula *T* = Ks/2λ, where Ks is peak Ks value and *λ* is the average substitution rate (6.5 × 10^−9^ substitutions/site/year for grasses; Gaut et al., 1996).

### Transcriptome-Based Gene Expression Analyses

The Illumina transcriptome sequencing data of six different organs of bermudagrass cultivar Yangjiang were aligned to the assembled genome using HISAT v2.1.1^48^ with default parameters (Kim et al., 2015). The numbers of mapped reads for each genes were converted to RPKM (reads per kilobase of transcript per million mapped fragments) values. The log_2_ transformed RPKM values were applied to perform Hierarchical clustering using Pearson’s correlation distance (Chen et al., 2021). The significantly expressed genes were defined as RPKM value > 1, the organ-enhanced genes were defined as RPKM value is 5-fold above the average RPKM values of other organs, whereas organ-enriched genes were defined as RPKM value is 5-fold above the RPKM values of any other organs (Uhlén et al., 2016; Nautiyal et al., 2020).

### Analyses of Homologous Recombination-Regulating Genes and Tiller-Angle-Regulating Genes

To obtain ZMM (acronym for Zip1-4, Msh4-5, and Mer3) protein-coding genes in *C. dactylon* and other 10 plant species, ZMM genes from *A. thaliana* were used as baits to search against the assembled genome of *C. dactylon* and other plant species recorded in Phytozome database or Ensembl Plants database^49^ using BLASTP v2.12.0 with an E-value cutoff of 10^−5^. The gene copy numbers and chromosome locations of different genes were manually summarized based on their identities. For *PROG1*, *LA1*, and *TAC1* genes, PROG1, LA1, and TAC1 proteins from *O. sativa* were used as baits to search against the assembled genome of *C. dactylon* and other seven species of *Oryza* genus recorded in Ensembl Plants database as described above. The amino acid sequences of proteins encoded by each gene families were searched against the Pfam database^50^ for domain comparisons (Mistry et al., 2021).

## Results

### Assembly of the *Cynodon dactylon* Genome

The *C. dactylon* cultivar Yangjiang was used for genome sequencing. As a national authorized *C. dactylon* cultivar., cultivar Yangjiang is a typical turf-type common bermudagrass and is widely used for turf planting in China (Zhang et al., 2018a; Supplementary Figure S1). Based on the K-mer genome survey result, the estimated genome size of *C. dactylon* cultivar Yangjiang is approximately 984 Mb, which is in line with the flow cytometry genome size estimation result of 1.02 Gb (Supplementary Figure S2). K-mer analysis also revealed that the genome of *C. dactylon* cultivar Yangjiang has a high heterozygosity (1.92%) with a repeat frequency of 56.91%.

**Figure 1:**
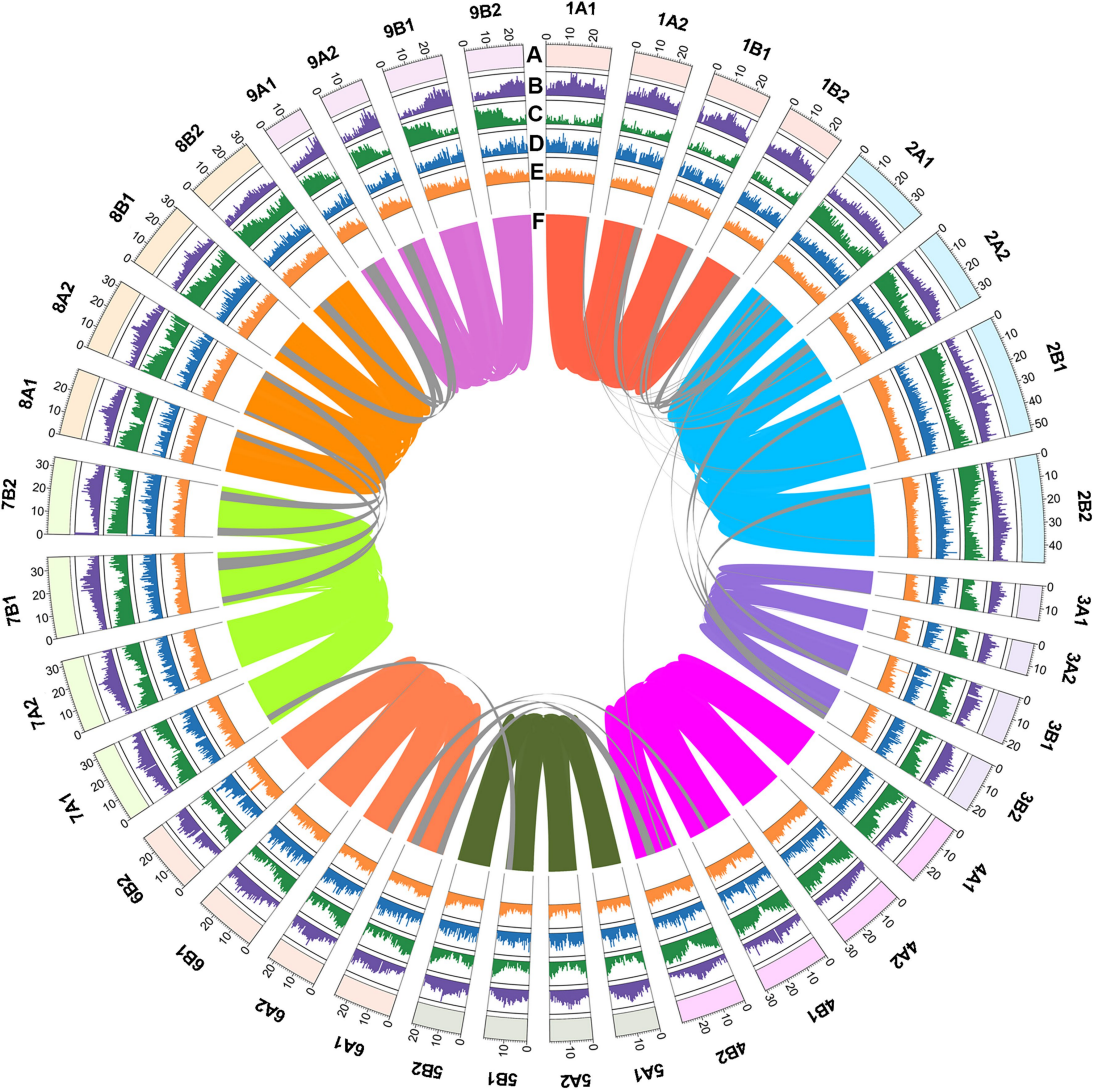
Genome features of *C. dactylon* cultivar Yangjiang. **(A)** Circular representation of the 36 pseudo chromosomes with scale mark labeling each 10 Mb. The density of **(B)** long-terminal repeat retrotransposons (LTR-RT), **(C)** protein-coding genes, **(D)** tandem repeat sequences, and **(E)** GC contents were calculated using 500 kb non-overlap window. **(F)** Inter-chromosomal synteny was illustrated with color lines.

To overcome the impact of high heterozygosity on the genome assembly, we adopted an integrated assembly strategy incorporating PacBio sequencing, Illumina sequencing, and Bionano and Hi-C techniques with the haplotype-resolving Hifiasm algorithm (Cheng et al., 2021; Supplementary Figure S3). Firstly, 151.99 Gb PacBio long reads (about 150× coverage of the genome) were *de novo* assembled into contigs, which were polished by 161.1 Gb Illumina paired-end reads (about 160× coverage of the genome; Supplementary Tables S1 and S2). Totally, 3,703 contigs with a N50 contig length of 2.65 Mb and a sum contig length of 1.295 Gb were obtained (Table 1). Secondly, 395.4 Gb Bionano optical maps (about 390× coverage of the genome) were used to integrate the contigs into scaffolds (Supplementary Figure S4; Supplementary Table S3). This procedure generated 241 scaffolds with a N50 scaffold length of 9.38 Mb and a sum scaffold length of 1.26 Gb (Table 1). Lastly, 231.3 Gb Hi-C data (24% useful information, about 55× coverage of the genome) were used to further cluster the scaffolds into pseudo chromosomes (Supplementary Figure S5; Supplementary Table S4). The finally obtained genome assembly (1.01 Gb) contained 36 chromosome-level superscaffolds, among which the longest and the shortest are 52.77 Mb and 14.32 Mb, respectively (Figure 1; Table 1). The assembly size was consistent with the estimated genome size. Furthermore, BUSCO analysis against the 1,375 *Embryophyta* gene sets indicated that 96.2% complete genes were successfully identified in the genome assembly, among which 88.1% were duplicated genes (Supplementary Table S5). These results collectively suggested that the assembled *C. dactylon* genome is high quality and complete.

**Table 1:** Statistics of *Cynodon dactylon* genome assembly.

	Illumina + PacBio	Illumina + PacBio + BioNano	Illumina + PacBio + BioNano + Hi-C
Assembly size (Mb)	1294.65	1258.08	1005.67
Scaffold number	3,703	241	36
N50 Scaffold length (Mb)	2.65	9.38	28.85
Longest scaffold (Mb)	13.42	34.64	52.77
Mean scaffold length (Mb)	0.35	5.22	27.94
Complete BUSCOs	97.80%	97.67%	96.20%

### Annotation of the *Cynodon dactylon* Genome

A total of 76,879 protein-coding genes with an average gene length of 3,535 bp and an average transcript number per gene of 1.9 were successfully predicted from the assembled genome (Table 2). The predicted gene model was also evaluated by BUSCO analysis. The result indicated that 1,324 (96.3%) complete core *Embryophyta* genes were identified and the majority (1,272, 96.07%) was duplicated genes (Supplementary Table S5). Among the predicted 146,743 transcripts, 87.89% (128, 966) were annotated by various functional database (Supplementary Figure S6; Supplementary Table S6). Functional classification further indicated that signal transduction mechanism, post-translation modification/protein turnover/chaperones, and transcription are the top three categories containing the largest number of transcripts (Supplementary Figure S7). Specifically, 4,888 transcription factors (TFs) belonging to 65 classes were successfully identified. Compared with other grass species, gene numbers of HSF, WRKY, NF-X1, NF-YA, NF-YC, CPP, GARP-G2-like, and DDT TF families were greatly increased in common bermudagrass (Supplementary Table S7). In addition, 6,265 non-protein-coding genes were also identified, including 1349 rRNAs, 2047 tRNAs, 1025 miRNAs, 1441 snoRNAs, and 403 snRNAs (Table 2).

**Table 2:** Statistics of *C. dactylon* genome annotation.

*Number of non-protein-coding genes*	6,264
Number of rRNA genes	1,349
Number of tRNA genes	2047
Number of miRNA genes	1,025
Number of snoRNA genes	1,340
Number of snRNA genes	503
*Number of protein-coding genes*	76,879
Mean gene length (bp)	3534.75
Percentage in genome (%)	27.02
Mean transcript number per gene	1.91
Total transcript number	146,743
Mean transcript length (bp)	1680.90
Mean 5′UTR length (bp)	145.36
Mean 3′UTR length (bp)	217.54
Mean coding sequence length (bp)	1392.22
Mean exon number per gene (bp)	7.29
Mean exon length (bp)	230.63
Mean intron number per gene (bp)	6.29
Mean intron length (bp)	409.82
*Number of repetitive sequences*	529,092
Mean repetitive sequence length (bp)	720.67
Percentage in genome (%)	37.91

**Figure 2:**
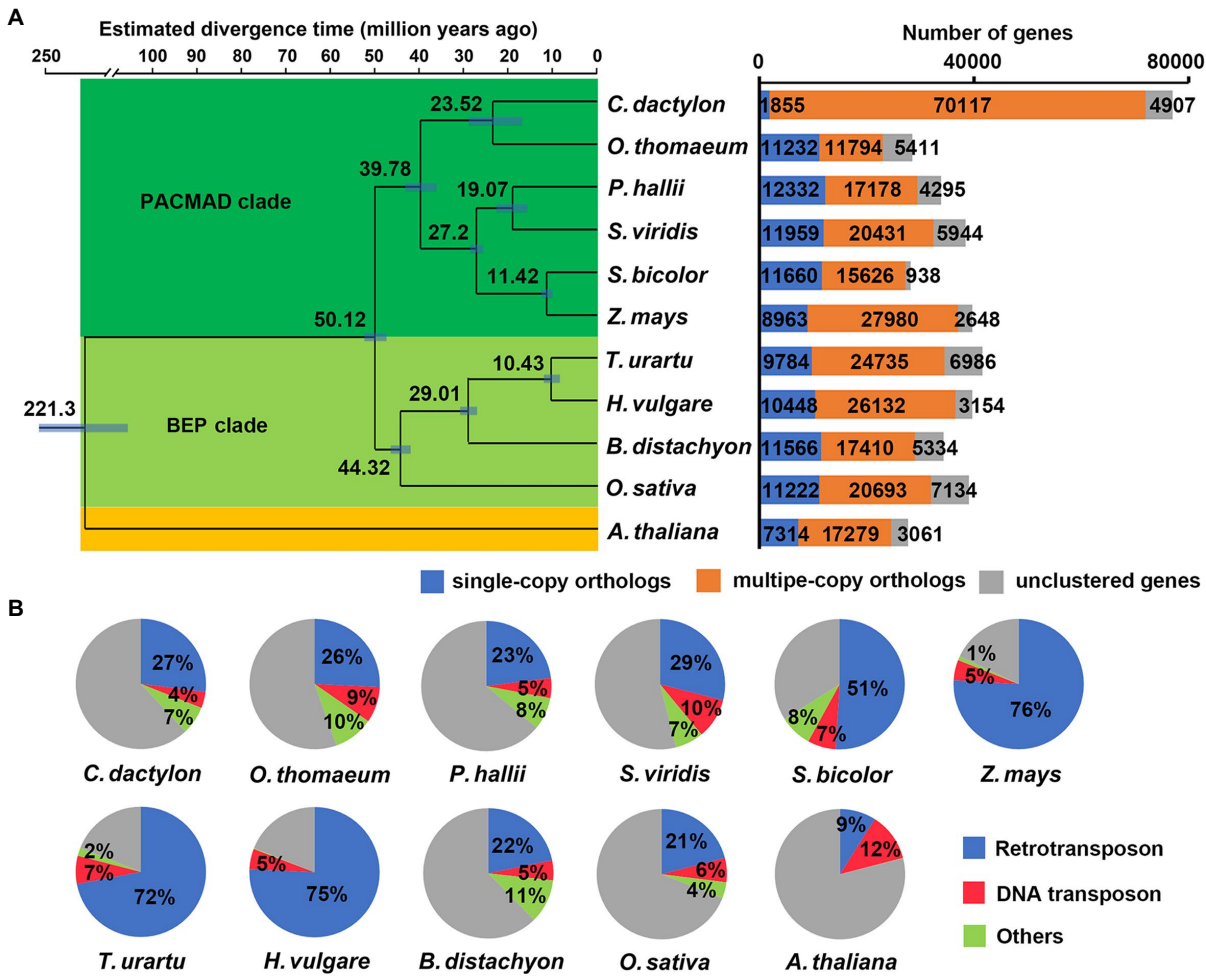
Comparative genomic analysis among *C. dactylon* and other plant species. **(A)** Phylogenetic relationship, divergence time, and gene family clustering of *C. dactylon* and other ten plant species. Left panel, Maximum parsimony (MP) species tree was constructed using protein sequences of 112 shared single-copy orthologous genes. The numbers in the brackets indicate the estimated divergence time of each node, and the blue bars show the 95% confidence interval of divergence time. All the nodes are 100% bootstrap support. Right panel, Orthologous gene families of *C. dactylon* and other ten plant species. **(B)** Comparison of repetitive sequences in *C. dactylon* and other ten plant species.

Orthologous clustering of protein-coding genes of *C. dactylon* with other ten plant species totally identified 32,695 orthologous gene families, including 7,792 commonly shared gene families and 3,173 bermudagrass-specific gene families consisting of 9,152 genes (Supplementary Tables S8 and S9). KOBAS enrichment analysis indicated that these bermudagrass-specific genes were enriched in glutathione metabolism, zeatin biosynthesis, ubiquitin mediated proteolysis, and other eight pathways (*q* value <0.05; Supplementary Table S10). In agreement with the BUSCO analysis result, orthologous gene clustering further revealed that as many as 91.2% (70117) of *C. dactylon* genes are members of 17,632 multiple-copy gene families, which is much higher than that of other ten species (Figure 2A; Supplementary Table S8). A phylogenetic tree was constructed based on the 112 shared single-copy orthologous genes (Figure 2A). The result indicated that *O. thomaeum* was closest to *C. dactylon* and the estimated divergence time of the two species was between 17.85 to 29.19 (midvalue of 23.52) million years ago (MYA). In line with phylogenic relationships, *C. dactylon* shared more orthologous gene families with members of the PACMAD (acronym for Panicoideae, Aristidoideae, Chloridoideae, Micrairoideae, Arundinoideae, and Danthonioideae) clade of grasses, including *O. thomaeum*, *S. bicolor*, and *S. viridis*, compared with *O. sativa* belonging to the BEP (acronym for Bambusoideae, Ehrhartoideae, and Pooideae) clade of grasses (Supplementary Figure S8).

A total of 381.3 Mb of repetitive sequences were also annotated in the assembled *C. dactylon* genome (Table 2 and Supplementary Table S11). The most abundant repetitive sequences are retrotransposons (70.95% of repetitive sequences and 26.9% of genome assembly), among which LTR-RTs and non-LTR-RT represent 84.73 and 15.27%, respectively. DNA transposons make up 11.29% of the repetitive sequences (4.28% of genome assembly), whereas tandem repeats and unclassified repeat sequences account for 2.79 and 3.75% of the assembled genome, respectively. Interestingly, the total repetitive sequence content and retrotransposon content in *C. dactylon* (37.91 and 26.9%, respectively) were similar to those of closely related species, including *O. thomaeum* (45 and 26%, respectively), *P. hallii* (36 and 23%, respectively), and *S. viridis* (46 and 29%, respectively), but much lower than those of distantly related species, including *Z. mays* (82 and 76%, respectively), *T. urartu* (81 and 72%, respectively), and *H. vulgare* (80 and 75%, respectively; Figure 2B). It is also noteworthy that genes are unevenly distributed in different chromosomes (39.87 to 104.80 Mb^−1^ in density), whereas similar distributions of repetitive sequences were found on all chromosomes of *C. dactylon* (482.23 to 559.93 Mb^−1^ in density; Supplementary Table S12). The annotated LTR-RTs were further used to calculate the LAI of the assembled genome. The total LAI score of *C. dactylon* genome is 13.63, implying that the current assembly of *C. dactylon* genome reached the reference genome level (Supplementary Table S13; Ou et al., 2018).

### Subgenome Composition of *Cynodon dactylon*

**Figure 3:**
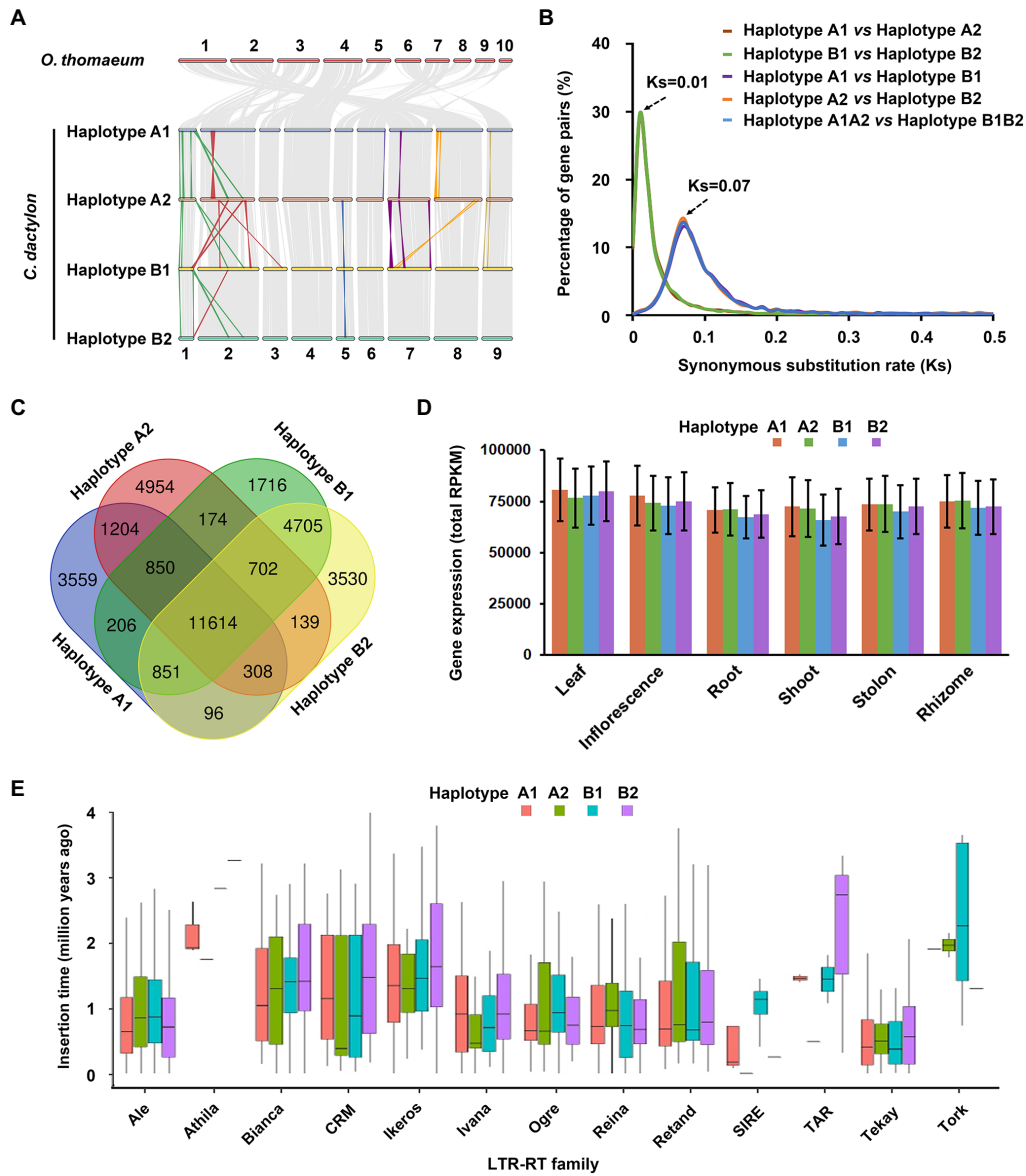
Subgenomic organization and variation of *C. dactylon.* **(A)** Schematic representation of syntenic genes among *O. thomaeum* and four haplotypes of *C. dactylon*. Gray lines depict homologous genome blocks. Color lines indicate inversion and translocation on the homologous chromosomes. **(B)** Distribution of synonymous nucleotide substitution levels (Ks) of syntenic gene pairs between different haploptypes of *C. dactylon.* **(C)** Venn diagram of alleles and orphan genes in the four haploptypes of *C. dactylon*. **(D)** Total gene expression level of the 11,614 four-copy alleles based on their relative expression level in six organs of *C. dactylon*. Error bars represent SE of the three sequencing replicates. **(E)** Box plots showing the insertion dynamics of 13 LTR-RT families in four haploptypes of *C. dactylon.* Box boundaries indicate the 25th and 75th percentiles of the insertion time and whiskers extend to 1.5 times the interquartile range.

Intra-genomic syntenic analysis totally detected 845 syntenic blocks containing 84,649 pairs of homoeologous genes in the *C. dactylon* genome, whereas 643 syntenic blocks containing 52,590 pairs of homoeologous genes were found between *C. dactylon* and *O. thomaeum* through inter-genomics syntenic analysis (Figure 3A and Supplementary Figure S9). Interestingly, the syntenic depth ratios of *C. dactylon* versus *O. thomaeum* and *C. dactylon* itself were 4:1 and 4:4, respectively, implying that *C. dactylon* genome is composed of four haplotypes containing the same number of chromosomes (Supplementary Figure S9). To distinguish homoeologous chromosomes from the four haplotypes of *C. dactylon*, putative centromeric array tandem repeat sequences were identified from the 36 chromosomes and were used to construct a maximum likelihood phylogenetic tree as previously described (Supplementary Table S14; VanBuren et al., 2020). The result indicated that the 36 centromeric array sequences showed distinguishing polymorphisms and could be clustered in four clades (Supplementary Figure S10). Based on this classification result and chromosome length variance, the four haplotypes, which were named as A1, A2, B1 and B2, respectively, were successfully resolved in the *C. dactylon* genome (Supplementary Table S15). In addition, syntenic analysis also revealed that chromosome 2, 3, and 10 of *O. thomaeum* are split and merged into chromosome 2 and 7 in four haplotypes of *C. dactylon*, whereas other chromosomes all have one-to-one correspondence (Figure 3A and Supplementary Figure S9).

Calculation of Ks of homologous gene pairs in the inter-genomic and intra-genomic synteny blocks not only confirmed the phylogenic analysis result that *C. dactylon* and *O. thomaeum* diverged at approximately 21.54 MYA (Ks = 0.28), but also indicated that two rounds of WGD events occurred in the evolutionary history of *C. dactylon* (Supplementary Figure S11). Specifically, the first WGD event occurred at approximately 5.38 MYA (Ks = 0.07), whereas the second WGD event occurred lately at about 0.77 MYA (Ks = 0.01; Supplementary Figure S11). Interestingly, the two WGD time points equivalent exactly to the divergence time of haplotypes A1/A2 with haplotypes B1/B2 and haplotype A1 with haplotype A2 (the same as haplotype B1 with haplotype B2), respectively (Figure 3B).

In combination with the orthologous gene clustering result, syntenic analysis totally identified 20,849 alleles in *C. dactylon* (Supplementary Table S16). Among these alleles, 11,614 have four allelic copies in all haplotypes, 2,711 have three allelic copies in three of four haplotypes, and 6,524 have two allelic copies in two of four haplotypes (Figure 3C and Supplementary Figure S12; Supplementary Table S17). Meanwhile, 3559, 4954, 1716, and 3530 orphan genes that exist as single-copy genes were also identified from haplotype A1, A2, B1, and B2, respectively (Figure 3C and Supplementary Figure S12; Supplementary Table S17). KOBAS enrichment analyses indicated that alleles were enriched in valine/leucine/isoleucine degradation, proteasome, brassinosteroid biosynthesis, and other eight pathways, whereas orphan genes were enriched in plant-pathogen interaction, base excision repair, DNA replication, and other nine pathways (q value <0.05; Supplementary Table S18). The expression abundance of alleles and orphan genes were further analyzed using the organ-specific transcriptome sequencing data of *C. dactylon* cultivar Yangjiang (Chen et al., 2021; Supplementary Table S19). The result indicated that similar portions of alleles and orphan genes in the four haplotypes were significantly expressed in six organs of bermudagrass; however, gene numbers of alleles and orphan genes enhance- or enrich-expressed in different organs, especially the three types of stems, varied greatly in the four haplotypes (Supplementary Figure S13; Supplementary Tables S20 and S21). Accordingly, the 11,614 four-copy alleles of the four haplotypes showed similar total expression abundance in the six organs (Figure 3D).

The distribution of repeat sequences in *C. dactylon* was also analyzed at the haplotype level. Among the four haplotypes, haplotype A2 and B1 has the minimum and maximum number of RTs, respectively (Supplementary Figure S14). By contrast, maximum number of four types of DNA transposons, including Tcl/mariner, EnSpm/CACTA, hAT, and muDR, was observed in haplotype B2, while haplotype A1 has the fewest muDR- and Helitron-type of DNA transposons (Supplementary Figure S14). Notably, total sequence length of Ty3-Gypsy LTR-RTs in haplotype B1 was 2.2 Mb larger than that of haplotype B2, which contributed approximately 40% of size variance between the two haplotypes, whereas another type of LTR-RTs, Ty1-Copia, showed similar sequence length in the two haplotypes (Supplementary Figure S14; Supplementary Tables S22 and S23). Moreover, 5,066 intact LTR-RTs were further used to estimate the insertion time of different families of LTR-RTs in *C. dactylon* genome (Supplementary Table S24). The results indicated that four families of LTR-RTs, including Athila, SIRE, TAR, and Tork, inserted into the four haplotypes of *C. dactylon* genome at different time, whereas other nine families showed similar insertion time range in the four haplotypes (Figure 3E). Interestingly, among the 244 active LTR-RTs with an insertion time of zero, 153 were located in three chromosomes of haplotype B1, 47 were located in two chromosomes of haplotype A1, whereas only 29 and 16 were located in single chromosome of haplotype A2 and B1, respectively (Supplementary Table S24).

### Adaptive Evolution of *Cynodon dactylon*

**Figure 4:**
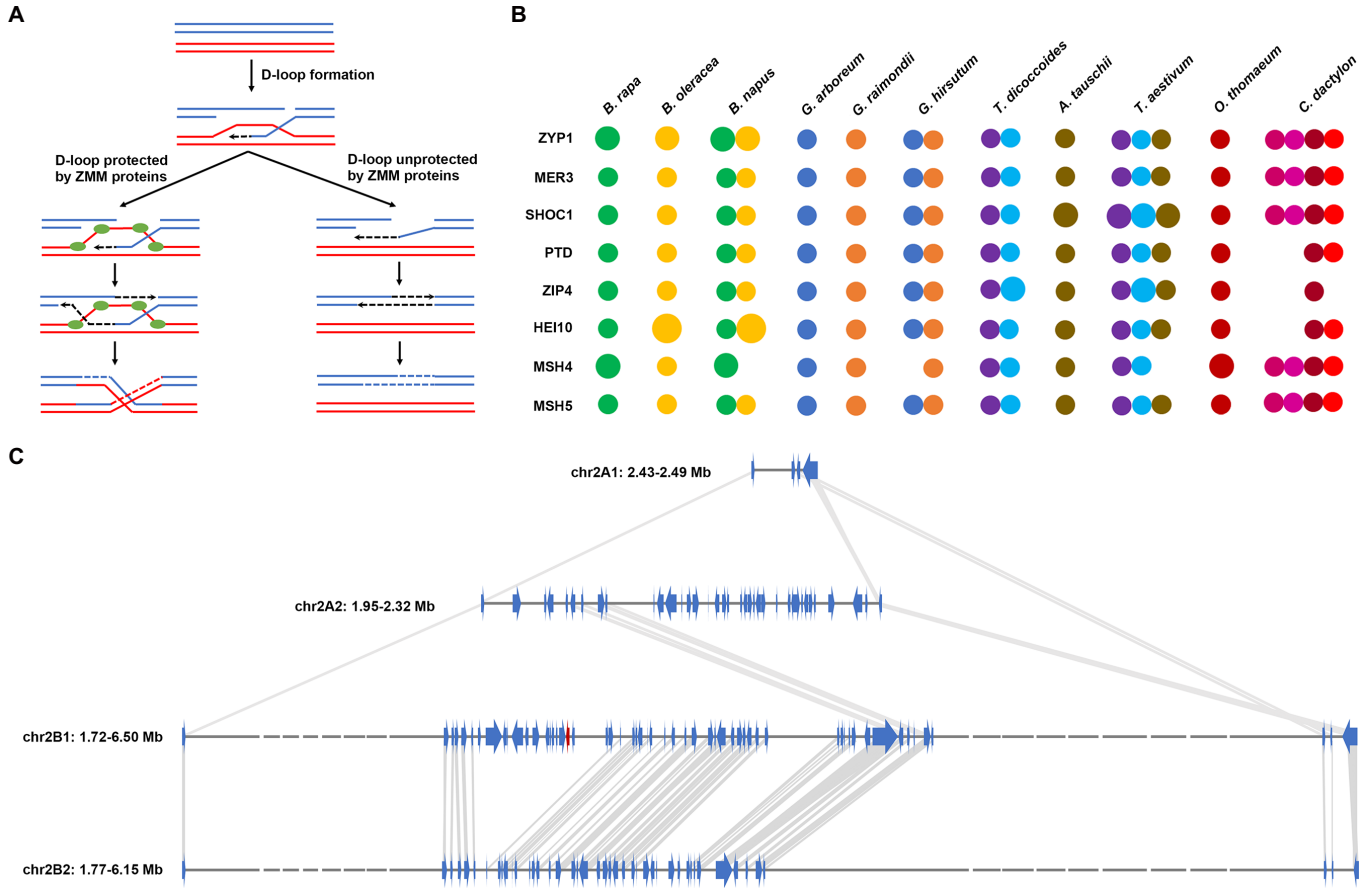
Evolution of homologous recombination-regulating genes in *C. dactylon.* **(A)** Diagram depicting the essential roles of ZMM proteins in regulating homologous recombination. **(B)** Comparison of the gene copy number of eight ZMM proteins in *C. dactylon*, *O. thomaeum*, and other nine species with different ploidy levels. **(C)** Enlarged chromosomal gene location map showing the loss of *ZIP4* gene and other contiguous genes in haplotype A1, A2, and B2 of *C. dactylon*. The *ZIP4* gene in haplotype B1 was shown in red color.

As a polyploid plant species with four sets of chromosomes, *C. dactylon* might develop a mechanism to control proper pairing and segregation of chromosomes during meiosis thus maintain its genome stability (Svačina et al., 2020). ZMM proteins, which stabilize the D-loop crossover intermediate of synapsis, are important homologous recombination regulators in all eukaryotes (Pyatnitskaya et al., 2019; Figure 4A). Previous studies have illustrated that gene copy number reduction of a ZMM protein, MSH4, could prevent meiotic crossovers between non-homologous chromosomes and stabilize the genome in allotetraploid *Brassica napus* (Gonzalo et al., 2019). Similar gene copy number reduction of MSH4 was also observed in other two polyploidy plants, allotetraploid *Gossypium hirsutum* and hexaploid *Triticum aestivum* (Figure 4B; Supplementary Table S25). However, *MSH4* and other four *ZMM* genes, including *ZYP1*, *MER3*, *SHOC1*, and *MSH5*, all existed as four-copy alleles in *C. dactylon* genome. By contrast, two *ZMM* genes, *PTD* and *HEL10*, existed as two-copy alleles, and another *ZMM* gene, *ZIP4*, existed as single-copy orphan gene in haplotype B1 of *C. dactylon* genome (Figure 4B; Supplementary Tables S25 and S26). Syntenic analysis further indicated that different sizes of chromosomal fragments containing *ZIP4* were lost in other three haplotypes (Figure 4C). These results collectively implied that *C. dactylon* also evolved a ZMM-dependent regulatory mechanism to maintain its genome stability as other polyploidy plants did; however, the key regulator might be ZIP4 rather than MSH4.

**Figure 5:**
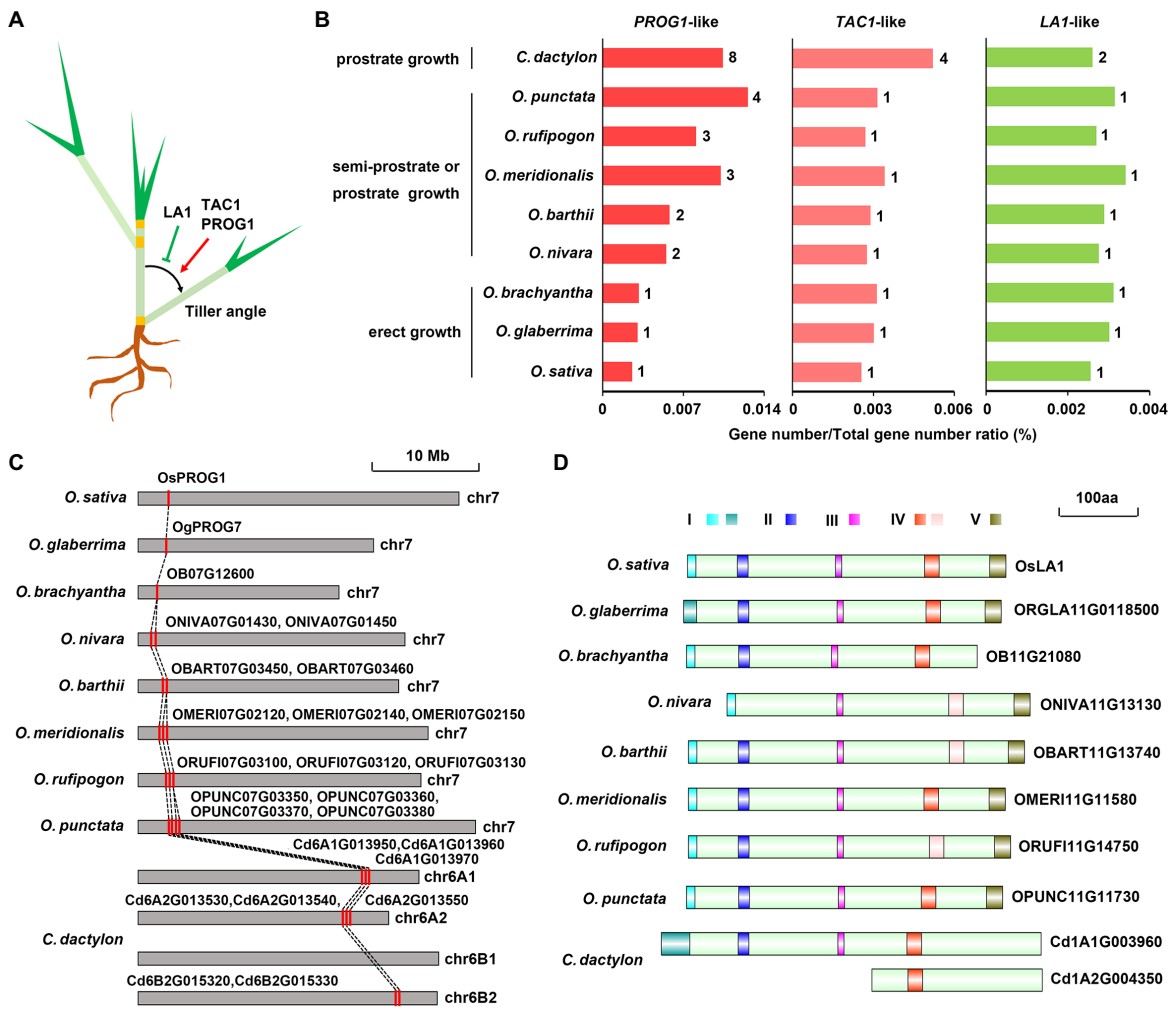
Evolution of tiller angle-regulating genes in *C. dactylon.* **(A)** Diagram depicting the positive/negative regulatory roles of *PROG1*, *LAZY1*, and *TAC1* genes in tiller angle control of grasses. **(B)** Comparison of the gene number of *PROG1*-, *LAZY1*-, and *TAC1*-like genes in *C. dactylon* and eight species of *Oryza* genus with different growth habits. **(C)** Syntenic relationship of *PROG1*-like genes in *C. dactylon* and eight species of *Oryza* genus with different growth habits. **(D)** Diagram showing the deficiency of key functional motifs in two LAZY1-like proteins of *C. dactylon* compared with those of eight species of *Oryza* genus.

As a widely used turfgrass species with two types of specialized stems, stolon and rhizome, *C. dactylon* exhibits a prostrate plant architecture owing to increased tiller angles of the two specialized stems (Dong and de Kroon, 1994). Previous studies have successfully identified several tiller-angle-regulating genes, including *PROG1*, *TAC1*, and *LA1*, in rice and other plants (Li et al., 2007; Yu et al., 2007; Jin et al., 2008; Tan et al., 2008; Figure 5A). Eight *PROG1*-like genes, four *TAC1*-like genes, and two *LA1*-like genes were also identified in *C. dactylon* (Figure 5B). Similar to semi-prostrate and prostrate growing *Oryza* genus plants, the family/genome gene number ratio of two prostrate growth-promoting genes, *PROG1*-like and *TAC1*-like, were higher than that of erect-growth-promoting *LA1*-like gene in *C. dactylon* (Figure 5B). Syntenic and phylogenic analysis revealed that six of the eight *PROG1*-like genes existed as three-copy alleles and the remaining two genes existed as two-copy alleles, the four *TAC1*-like genes existed as four-copy alleles, whereas two *LA1*-like genes existed as two-copy alleles (Figure 5C and Supplementary Figures S15, S16). Pfam domain analysis further indicated that all eight PROG1-like proteins have the conserved C_2_H_2_-type zinc finger domain identified in the functional OsPROG1 and OgPROG7 proteins, however, both two LA1-like proteins of *C. dactylon* lack the functional C-terminal conserved region V (Figure 5D and Supplementary Figure S15). Moreover, the LA1-like protein encoded by the allele of haplotype A2 further lack the functional N-terminal conserved region I and two other conserved regions II and III (Yoshihara and Spalding, 2020; Figure 5D). In combination with the observation that large chromosome fragments containing the *LA1-like* gene locus were lost in other two haplotypes (Supplementary Figure S16), sequence variation of *LA1-like* genes in the two residual alleles suggested that LA1 protein activity was inhibited in *C. dactylon*. In addition, both two *LA1*-like genes were weakly expressed in stolon and rhizome, whereas three of four *TAC1*-like genes were preferentially expressed in the two specialized stems (Supplementary Figure S16; Supplementary Table S27). These results collectively implied that different tiller-angle-regulating genes were synergistically evolved to promote a prostrate plant architecture in *C. dactylon*.

## Discussion

Turfgrasses are important groups of grass species serving essential functions, including soil stabilization, water conservation, filtration of air, and water borne pollutants, in urban and suburban landscapes (Huang, 2021). In the past several years, genome sequences of many turfgrass species, including zoysiagrasses (*Zoysia japonica* and *Zoysia matrella*), perennial ryegrass (*Lolium perenne*), centipedegrass (*Eremochloa ophiuroides*), and African bermudagrass (*C. transvaalensis*), were successfully sequenced and assembled using different techniques (Tanaka et al., 2016; Cui et al., 2021; Frei et al., 2021; Wang et al., 2021). In this study, we reported a high-quality haplotype-resolved genome of another important turfgrass species, common bermudagrass (*C. dactylon*), consisting of 36 pseudo chromosomes with a contig N50 of 2.65 Mb and a LAI score of 13.63 (Figure 1; Table 1 and Supplementary Table S13). The assembled nome of *C. dactylon* not only offers a solid foundation to study the molecular basis of valuable agronomic traits as well as molecular breeding of this important turfgrass species, but also provides an essential resource for comparative genomic analysis among different turfgrasses and other grasses.

The most prominent characteristics of *C. dactylon* genome are the presence of four haplotypes, named as A1, A2, B1, and B2, respectively. As an allotetraploid plants with high heterozygosity (1.92%), *C. dactylon* has four sets of chromosomes with significant differences that could be discriminated as different haplotypes using the newest haplotype-resolving Hifiasm algorithm; thus, an A1A2B1B2 genome assembly with 36 chromosomes, rather than an AB genome assembly with 18 chromosomes, was finally obtained (Kyriakidou et al., 2018). Similar result was also observed in the haplotype-phased genome assembly of tetraploid blueberry (2n = 4x = 48), which also reported a four haplotype-resolved genome containing 48 pseudo chromosomes (Colle et al., 2019). Notably, all the four haplotypes of *C. dactylon* have nine chromosomes; however, the total chromosome size showed variance among different haplotypes (Figure 3A; Supplementary Table S15). Specifically, haplotype A1 and A2 have a similar size (236.96 Mb and 231.36 Mb), whereas haplotype B1 and B2 have another similar size (271.19 Mb and 266.17 Mb; Supplementary Table S15). Accordingly, genes and repeat sequences in haplotype A1 and A2 are fewer than those in haplotype B1 and B2 (Supplementary Table S15). Interestingly, the size of four haplotypes are similar to the genome size of *O. thomaeum* (10 chromosomes, 243 Mb) and the monoploid genome size of *Eragrostis tef* (10 chromosomes, 288 Mb), two grass species belonging to the Chloridoideae subfamily of PACMAD clade of grasses as *C. dactylon* does, but much smaller than the genome size of African bermudagrass *C. transvaalensis* (nine chromosomes, 444 Mb), which is classified along with *C. dactylon* in the same *Cynodon* genus (VanBuren et al., 2015, 2020; Cui et al., 2021). Similar chromosome size variation was also observed between *Morus notabilis* and *M. alba*, both of which belongs to the same *Morus* genus (Xuan et al., 2022). These findings collectively suggested that genome size variation among different plant species might not be simply correlated with their phylogenic relationships.

Whole-genome duplication is an extreme mechanism of gene duplication that leads to a sudden increase in both genome size and the entire gene set thus plays important roles in plant genome evolution (Clark and Donoghue, 2018). Ks analysis revealed that two rounds of WGD events occurred in *C. dactylon*, which is in correspondence to the divergence time of haplotypes A1/A2 with haplotypes B1/B2 at 5.38 MYA and haplotype A1 with haplotype A2 at 0.77 MYA (the same as haplotype B1 with haplotype B2), respectively (Figure 3B and Supplementary Figure S11). These results collectively implied a complex evolutionary history of *C. dactylon*. At approximately 5.38 MYA, the ancestor of haplotype A1 and A2, named as A, might hybridized with B, the ancestor of haplotype B1 and B2, to form an AB hybrid species. At about 0.77 MYA, either an autopolyploidization event occurred in the AB hybrid species that doubled the genome to AABB or a secondary hybridization event occurred between two AB hybrid species to form an ABAB hybrid species through allopolyploidization, both of which could finally evolved into the present A1A2B1B2 genome of *C. dactylon*. The latter allopolyplodization mechanism seems more possible because the ratio of coupling to repulsion linkage phase of nondistorted mapped loci was approximately 1: 1 in an SSR-maker based linkage mapping of the first-generation selfed population of *C. dactylon* (Guo et al., 2017). Similar two rounds of WGD events were also observed in the formation of the polyploidy genome of *Miscanthus floridulus* and *Saccharum spontaneum*, suggesting a conserved evolution mechanism might exist in different genus of polyploid grasses (Zhang et al., 2018b, 2021).

A dominant subgenome often emerges immediately following the WGD event in the genome of allopolyploids (Liang and Schnable, 2018). However, some recent allopolyploids, including the above-mentioned *M. floridulus* and *S. spontaneum*, display indistinguishable or slight subgenome dominance (Zhang et al., 2018b, 2021). Orthologous gene clustering analysis indicated that four haplotypes of *C. dactylon* shared similar number of gene families with *O. thomaeum* (Supplementary Figure S8). Syntenic analysis further revealed that the four haplotypes have 12,197 (68.50% of 17,805), 12,039 (68.40% of 17,600), 14,406 (69.20% of 20,818), and 14,347 (69.46% of 20,656) syntenic orthologs to *O. thomaeum*, respectively (Supplementary Figure S9). These results suggested that four subgenomes of *C. dactylon* did not experience biased gene loss during evolution. Moreover, although a few genes from different haplotypes showed biased expression in different organs, overall gene expression levels showed high similarity among the four haplotypes (Figure 3D and Supplementary Figure S13). In addition, similar distribution and insertion time of LTRs were also observed in the four haplotypes (Figure 3E and Supplementary Figure S14). Taken together, these analyses collectively implied subgenome dominance is also unobvious in *C. dactylon*.

Polyploidy brings many advantages to polyploid plants. Heterosis could foster a greater biomass and accelerated development, whereas gene redundancy could mask deleterious mutations and diversify the functions of extra gene copies (Comai, 2005). As a worldwidely distributed grass species inhabiting diverse and harsh environments, allotetraploid *C. dactylon* undoubtedly benefits from these advantages. However, long-term survival of polyploid plants also require a mechanism to withstand the extensive genomic instability that accompanies with the presence of multiple pairing chromosomes in meiosis (Mason and Mason and Wendel, 2020). As a clonal plant with stolons and rhizomes, *C. dactylon* reproduces asexually through regenerating new plants from axillary buds of stolon and rhizome node (Dong and de Kroon, 1994), thus bypasses meiosis and recombination in gamete generation process. On the other hand, a ZMM-dependent regulatory mechanism to maintain genome stability during meiosis was also identified in *C. dactylon* (Figure 4). Owing to these belt and braces strategies, four unbiased haplotypes of subgenome are stably maintained in *C. dactylon* genome.

Tiller angle (branch angle in eudicot plants) is an important plant architectural trait affecting the density of growing plants (Wang et al., 2022). Cereal grasses often have compact and erect plant architecture characteristics with small tiller angles, which is essential for high yields. Specifically, successful domestication of cultivated rice from wild rice ancestors depended on the transition from prostrate growth to erect growth, in which process the tiller angle was greatly reduced (Li et al., 2007; Yu et al., 2007; Jin et al., 2008; Tan et al., 2008). However, for turfgrasses including *C. dactylon*, prostrate growth mode with large tiller angle is more preferable because it could accelerate turf formation, increase soil coverage, and diminish mowing frequency (Wang et al., 2021). Blast searches indicated that key tiller-angle-regulating genes reported in rice and other plants, including *PROG1*, *LA1*, and *TAC1*, were highly conserved in *C. dactylon* (Figure 5B). Similar to prostrate growing wild rice species, clustering of *PROG1*-like C_2_H_2_ transcription factor genes in adjacent positions of chromosomes were observed in *C. dactylon* (Wu et al., 2018; Huang et al., 2020; Figure 5C and Supplementary Figure S15). By contrast, *LA1*-like genes that promote erect growth not only experienced gene copy lost due to large chromosomal fragment deletions but also mutated to form truncated proteins (Figure 5D and Supplementary Figure S16). These results strongly suggested that similar selection pressure might also exist in *C. dactylon* to form the prostrate plant architecture characteristics as the domestication of rice from wild rice; however, the selection target might be *LA1* rather than *PROG1*.

## Conclusion

The genome of a widely used warm-season turfgrass species, *C. dactylon*, was sequenced and annotated in this study. The assembled genome contains 36 pseudo chromosomes, includes 37.91% genome size of repeat sequences, and encodes 76,879 protein-coding genes. The polyploid *C. dactylon* genome is consists of four haplotypes derived from two rounds of WGD events. Although a few haplotype-specific genes and transposons were identified, no global subgenome dominance was detected among the four haplotypes. A ZMM-dependent regulatory mechanism to maintain the genome stability was successfully identified. Furthermore, synergistic evolution of tiller-angle-regulating genes was also observed. In summary, the extensive datasets and analyses presented in this study not only offer an essential resource for basic studies and breeding researches of turfgrasses, but also provide new insights into regulation mechanisms underlying polyploid genome stability and prostrate growth.

## Data Availability Statement

The datasets presented in this study can be found in online repositories. The names of the repository/repositories and accession number(s) can be found at: National Center for Biotechnology Information (NCBI) BioProject database under accession numbers PRJNA430136, PRJNA685207, and PRJNA805105.

## Author Contributions

BZ and J-YL planned and managed the project and wrote the manuscript. J-YL provided the research fund. BZ, SC, JC, and DL conducted the research and analyzed the data. JL provided the plant material and helped to write the manuscript. Y-BY helped to analyze the data and write the manuscript. All authors contributed to the article and approved the submitted version.

## Funding

This work was financially supported by the Science and Technology Development Foundation of Tsinghua University.

## Conflict of Interest

The authors declare that the research was conducted in the absence of any commercial or financial relationships that could be construed as a potential conflict of interest.

## Publisher’s Note

All claims expressed in this article are solely those of the authors and do not necessarily represent those of their affiliated organizations, or those of the publisher, the editors and the reviewers. Any product that may be evaluated in this article, or claim that may be made by its manufacturer, is not guaranteed or endorsed by the publisher.

## Footnotes

^1^ https://github.com/BGI-flexlab/SOAPnuke

^2^ https://github.com/gmarcais/Jellyfish

^3^ http://qb.cshl.edu/genomescope

^4^ https://github.com/chhylp123/hifiasm

^5^ https://github.com/isovic/racon

^6^ https://github.com/bwa-mem2/bwa-mem2

^7^ https://github.com/broadinstitute/pilon

^8^ https://bionanogenomics.com/support/software-downloads

^9^ https://github.com/aidenlab/juicer

^10^ https://github.com/aidenlab/3d-dna

^11^ https://www.girinst.org/server/RepBase

^12^ http://www.repeatmasker.org

^13^ http://www.repeatmasker.org/RepeatModeler

^14^ http://www.drive5.com/piler

^15^ http://bix.ucsd.edu/repeatscout

^16^ http://tlife.fudan.edu.cn/tlife/ltr_finder

^17^ https://tandem.bu.edu/trf/trf.html

^18^ https://github.com/oushujun/LTR_retriever

^19^ https://phytozome-next.jgi.doe.gov

^20^ https://www.ebi.ac.uk/Tools/psa/genewise

^21^ https://github.com/Gaius-Augustus/Augustus

^22^ https://genome.crg.es/software/geneid/

^23^ http://www.softberry.com

^24^ http://ccb.jhu.edu/software/glimmerhmm

^25^ http://argonaute.mit.edu/GENSCAN.html

^26^ http://research-pub.gene.com/gmap

^27^ https://anaconda.org/bioconda/pasa

^28^ http://ccb.jhu.edu/software/tophat

^29^ http://cole-trapnell-lab.github.io/cufflinks

^30^ http://evidencemodeler.github.io

^31^ http://eggnog5.embl.de

^32^ http://eggnog-mapper.embl.de

^33^ http://www.geneontology.org

^34^ http://www.genome.jp/kegg

^35^ http://bioinfo.org/kobas

^36^ http://bioinfo.bti.cornell.edu/tool/itak

^37^ http://planttfdb.gao-lab.org

^38^ https://github.com/tseemann/barrnap

^39^ http://trna.ucsc.edu/tRNAscan-SE

^40^ http://eddylab.org/infernal

^41^ https://busco.ezlab.org

^42^ https://github.com/davidemms/OrthoFinder

^43^ https://www.ebi.ac.uk/Tools/msa/muscle

^44^ http://abacus.gene.ucl.ac.uk/software/

^45^ http://www.timetree.org

^46^ https://ftp.ncbi.nlm.nih.gov/blast/executables/

^47^ https://github.com/wyp1125/MCScanX

^48^ http://daehwankimlab.github.io/hisat2

^49^ http://plants.ensembl.org

^50^ https://pfam.xfam.org
