# Supplementary material for: A High-Quality Haplotype-Resolved Genome of Common Bermudagrass (Cynodon dactylon L.) Provides Insights Into Polyploid Genome Stability and Prostrate Growth
Source: Front Plant Sci. 2022 Apr 25;13:890980. doi: 10.3389/fpls.2022.890980 (PMC9081840; doi:10.3389/fpls.2022.890980)
Supplement: Supplementary file 1 [file Data_Sheet_1.DOCX]

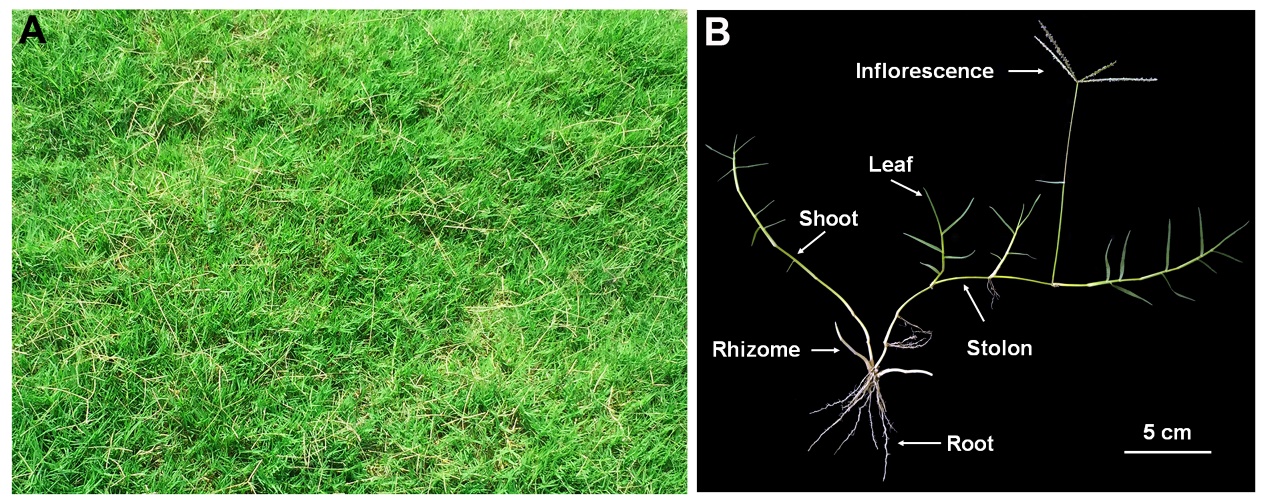
**Figure S1. Plant characteristics of *C. dactylon* cultivar Yangjiang**

**A.** Compact and uniform turf formed through the clonal growth of *C. dactylon* cultivar Yangjiang. **B.** Coexist of three types of stems (shoot, stolon and rhizome) in plant of *C. dactylon* cultivar Yangjiang.


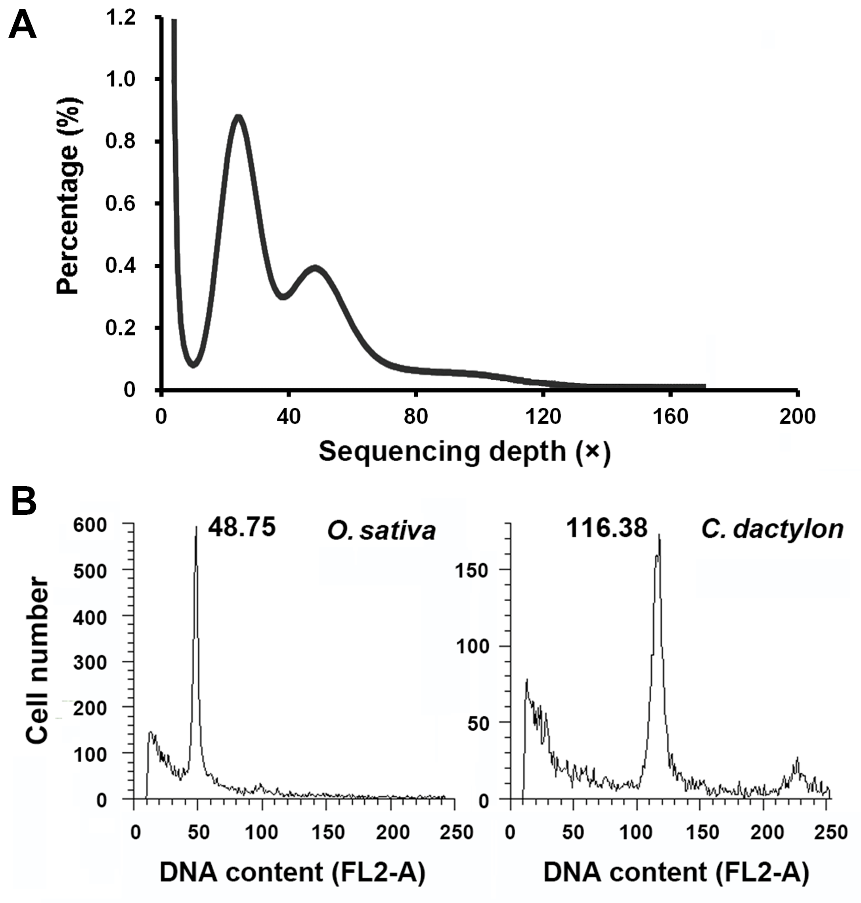


**Figure S2. Estimation of the genome size of *C.* *dactylon* cultivar Yangjiang**

**A.** K-mer genome survey of *C. dactylon* cultivar Yangjiang. **B.** Flow cytometry analysis of the genome size of *C. dactylon* cultivar Yangjiang with *O. sativa* as internal reference.





**Figure S3. Strategy to sequence the *C. dactylon* genome**


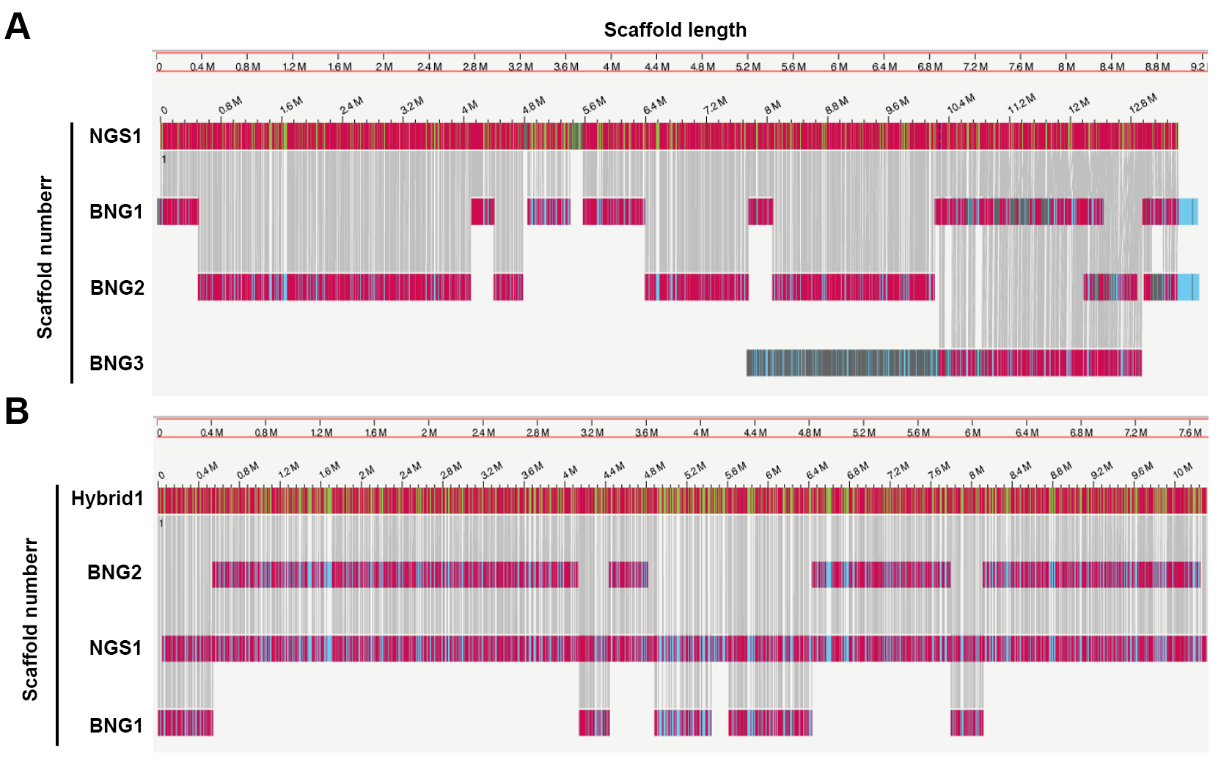


**Figure S4. BioNano-assisted genome assembly of *C.* *dactylon***

**A.** Error correction of the assembled scaffold NGS1 using BioNano optical maps BNG1, 2 and 3. **B.** Construction of the hybrid scaffold Hybrid1 using the assembled scaffold NGS1 and BioNano optical maps BNG1 and 2.


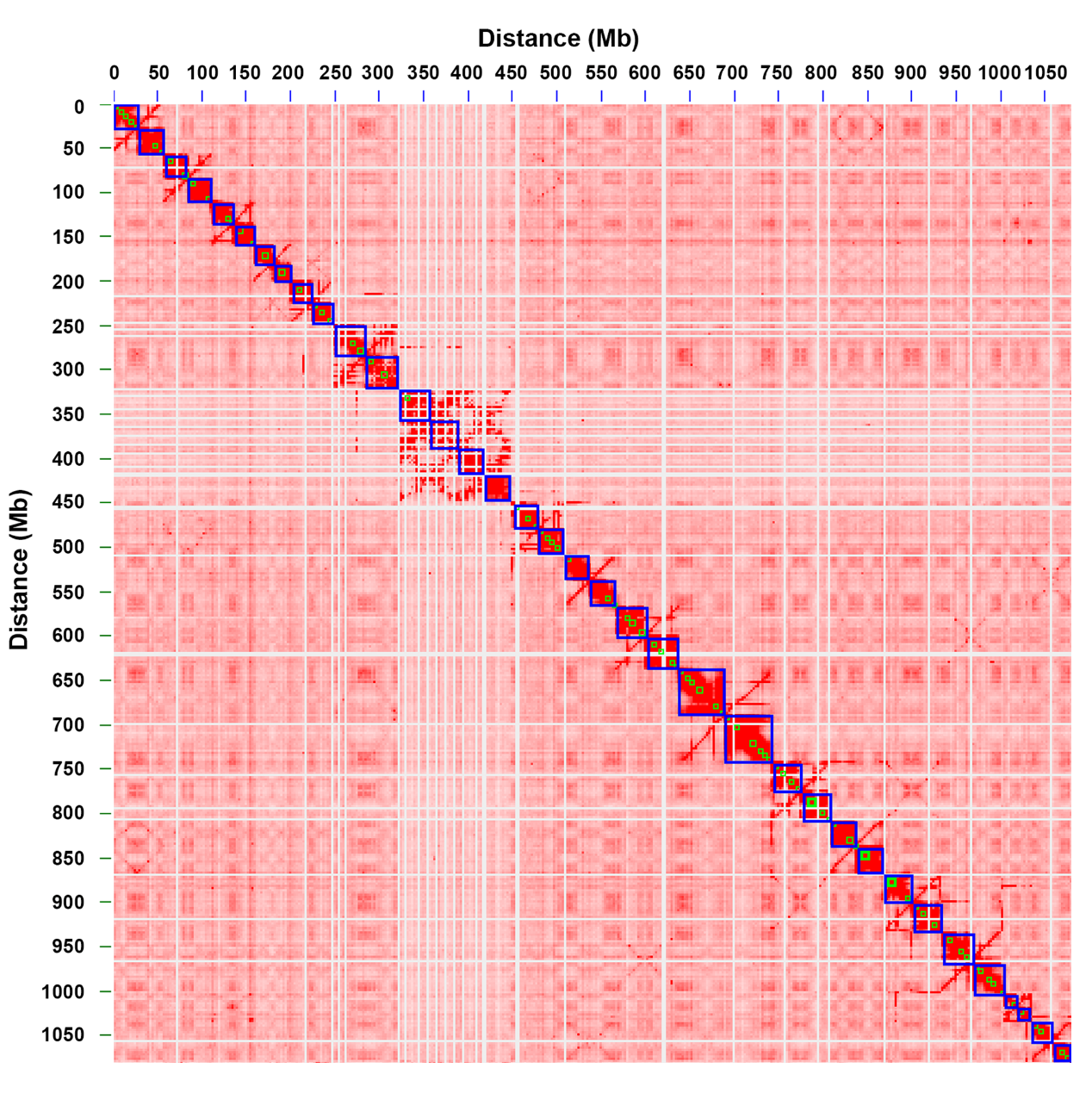


**Figure S5. Hi-C heatmap showing interactions among the 36 pseudo chromosomes of *C. dactylon***

**
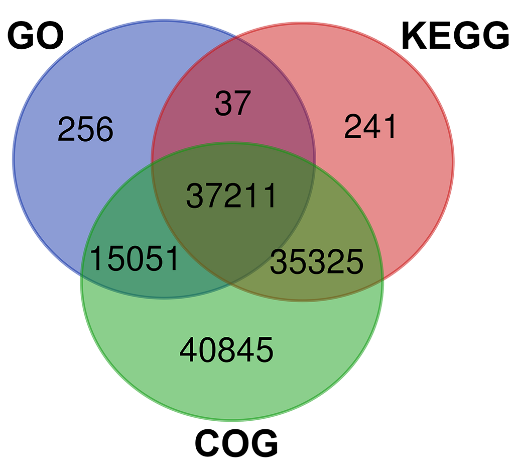
**

**Figure S6. Venn diagram of GO, KEGG and COG annotation results of predicted proteins in *C. dactylon***


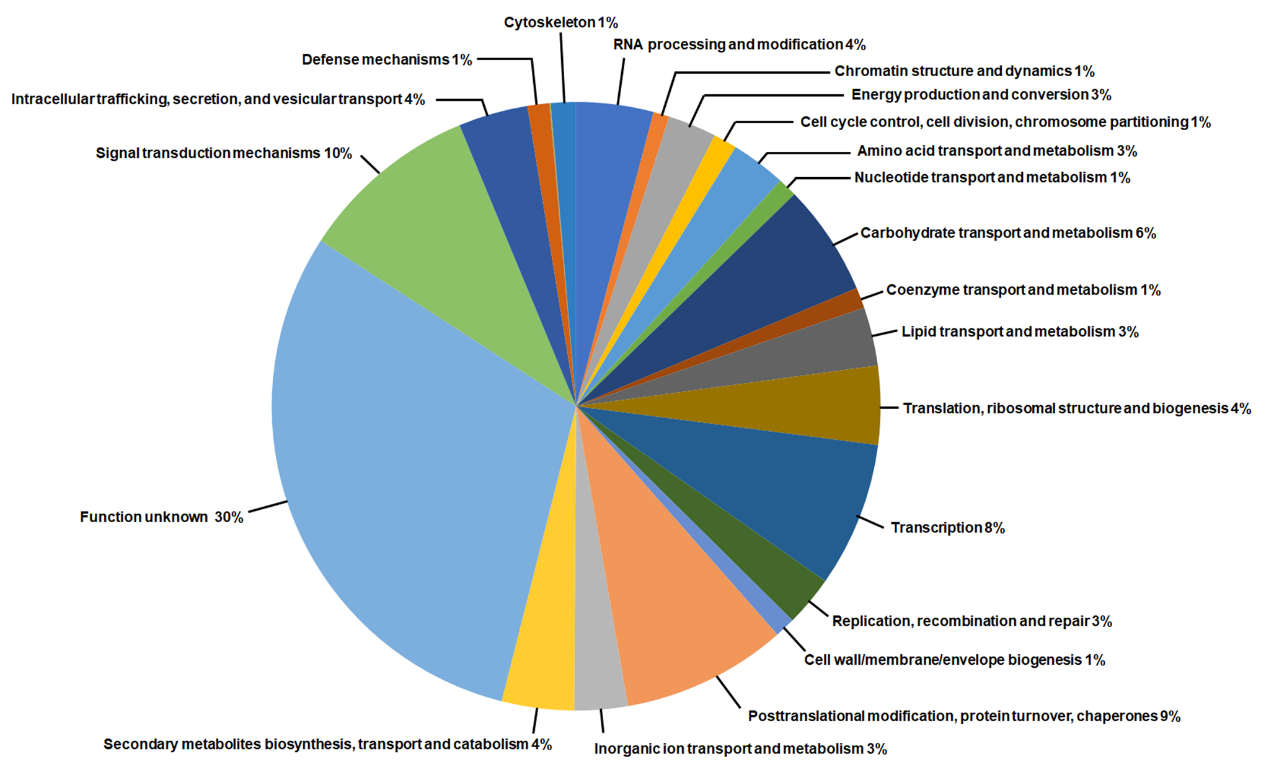


**Figure S7. Functional classification of predicted proteins in *C. dactylon***


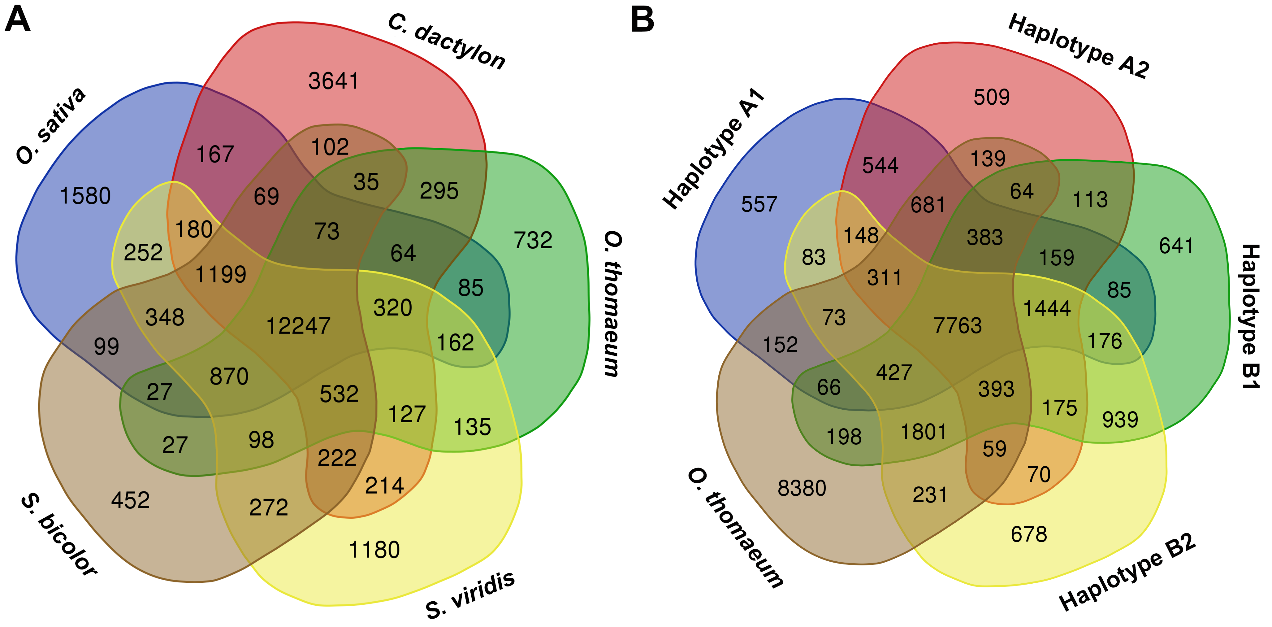


**Figure S8. Intra-genomic and inter-genomic gene family analyses of *C. dactylon***

**A.** Venn diagram of the shared and unique gene families among *C. dactylon*, *O. thomaeum*, *S. viridis*, *S. bicolor* and *O. sativa*. **B.** Venn diagram of the shared and unique gene families among *O. thomaeum* and four haplotypes of *C. dactylon*.


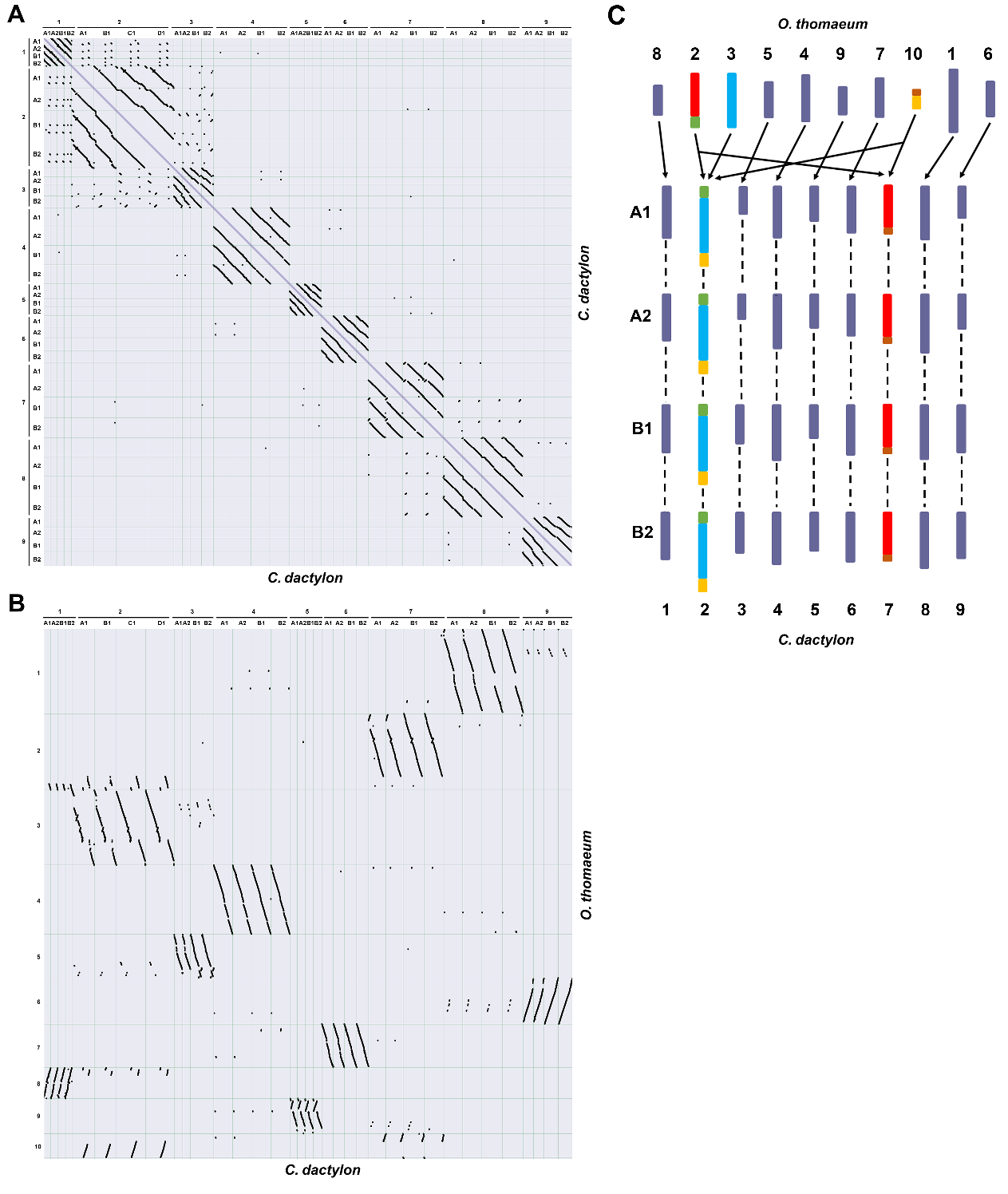


**Figure S9. Macrosynteny analysis of the *C. dactylon* genome**

**A.** Macrosynteny of the four haplotypes of *C. dactylon*. **B.** Macrosynteny of the four haplotypes of *C. dactylon* with *O. thomaeum*. **C.** Chromosome evolution from *O. thomaeum* to *C. dactylon* revealed by the synteny analysis.


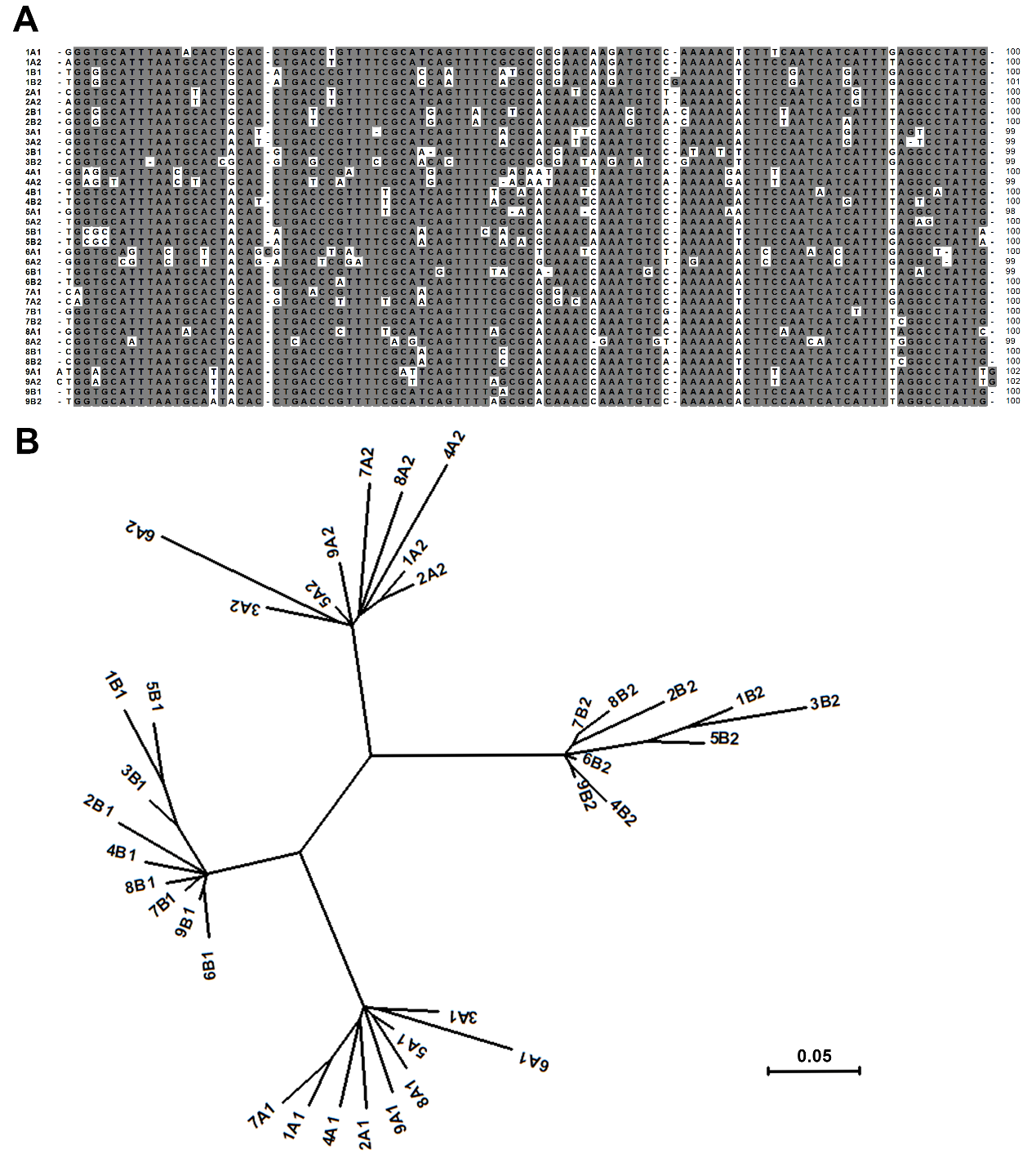


**Figure S10. Classification of centromeric repeat arrays in *C. dactylon***

**A.** Alignment of the centromeric repeat array sequences in *C. dactylon*. A 100 bp subset of the array sequences with high homology is shown. **B.** Maximum likelihood phylogenetic tree of the centromeric repeat array sequences in *C. dactylon*.


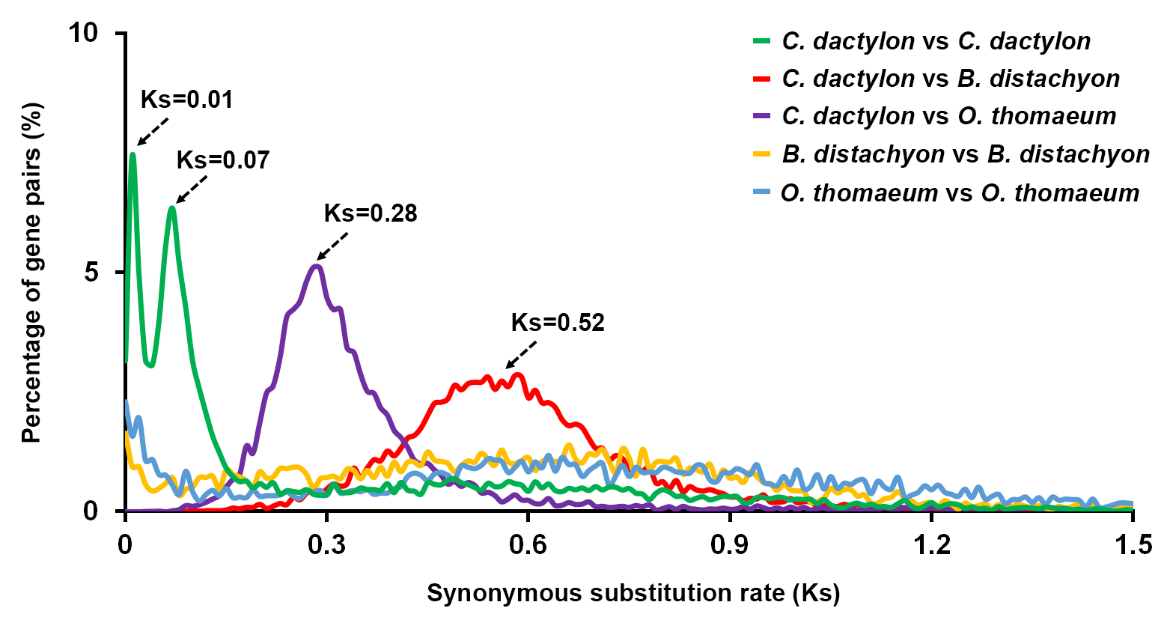


**Figure S11. Ks distributions of homologous gene pairs in the genomes of *C. dactylon*, *O. thomaeum* and *B. distachyon***


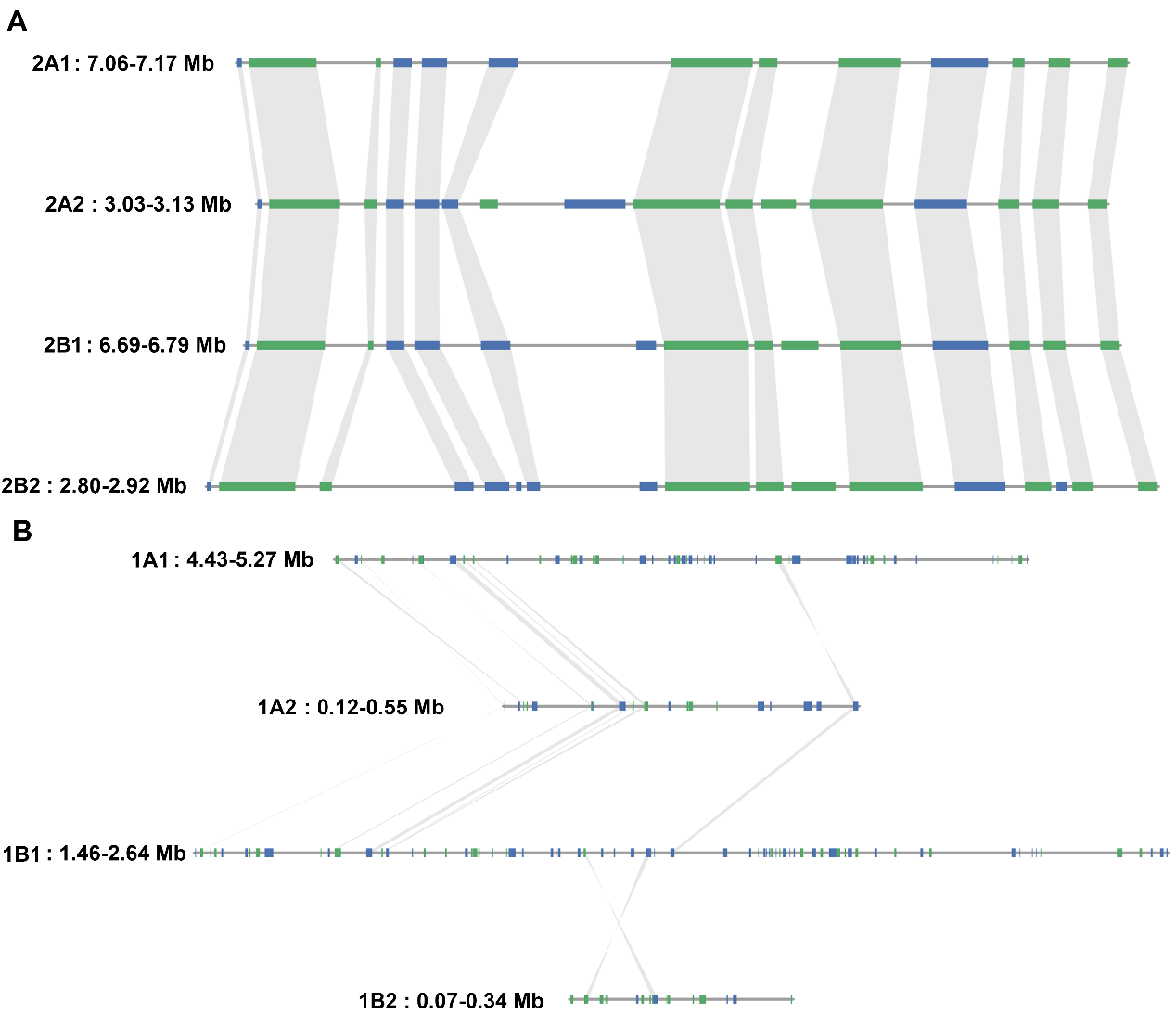


**Figure S12. Microsynteny analysis of the *C. dactylon* genome segments**

**A.** Tightly colinearity of a 0.1 Mb region of chromosome 2 in four haplotypes of *C. dactylon* showing the existence of 13 four-copy alleles and 4 orphan genes. **B.** Poorly colinearity of a 0.5 Mb region of chromosome 1 in four haplotypes of *C. dactylon* showing the existence of 6 three-copy alleles, 3 two-copy alleles and 130 orphan genes.

**
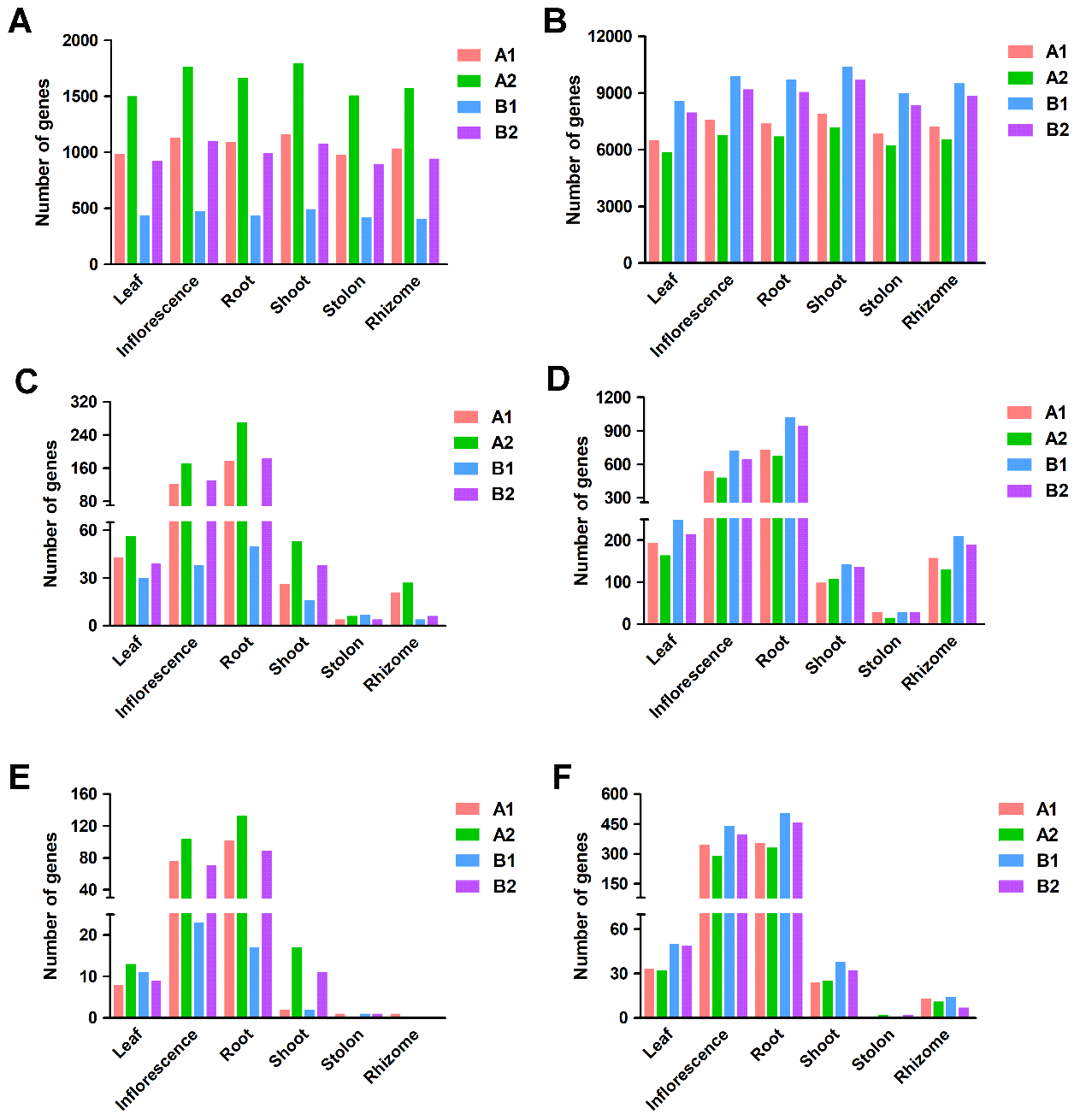
**

**Figure S13. Organ expression bias in the four haploptypes of *C. dactylon***

**A-B.** Distribution of significantly expressed **A.** orphan genes and **B.** alleles of the four haplotypes in six organs. **C-D.** Distribution of organ-enhance expressed **C.** orphan genes and **D.** alleles of the four haplotypes in six organs. **E-F.** Distribution of organ-enrich expressed **E.** orphan genes and **F.** alleles of the four haplotypes in six organs.

**
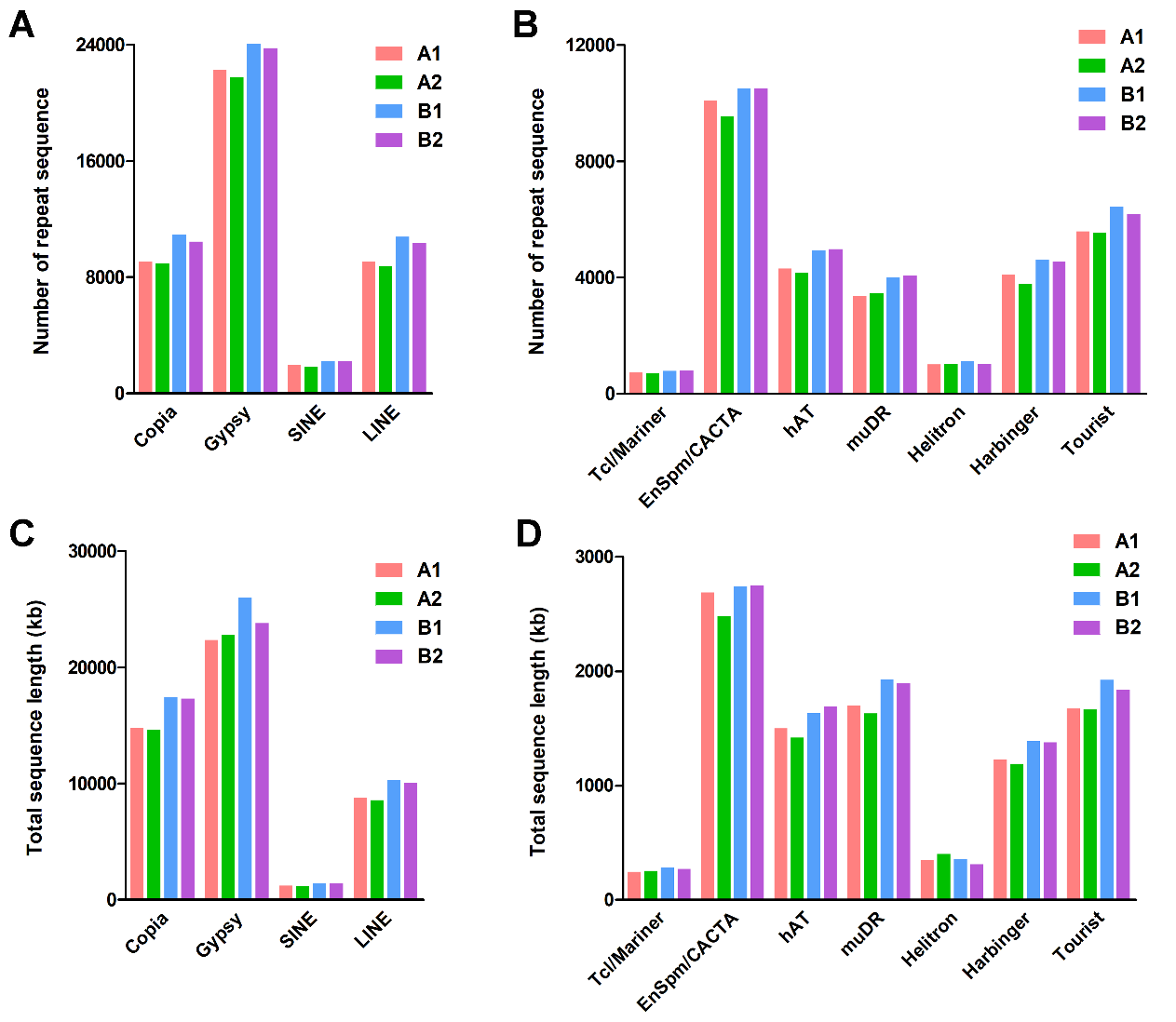
**

**Figure S14. Distribution of repeat sequences in four haplotypes of *C. dactylon***

**A.** Number of different classes of retrotransposons in four haplotypes. **B.** Number of different classes of DNA transposons in four haplotypes. **C.** Sequence length of different classes of retrotransposons in four haplotypes. **D.** Sequence length of different classes of DNA transposons in four haplotypes.

**
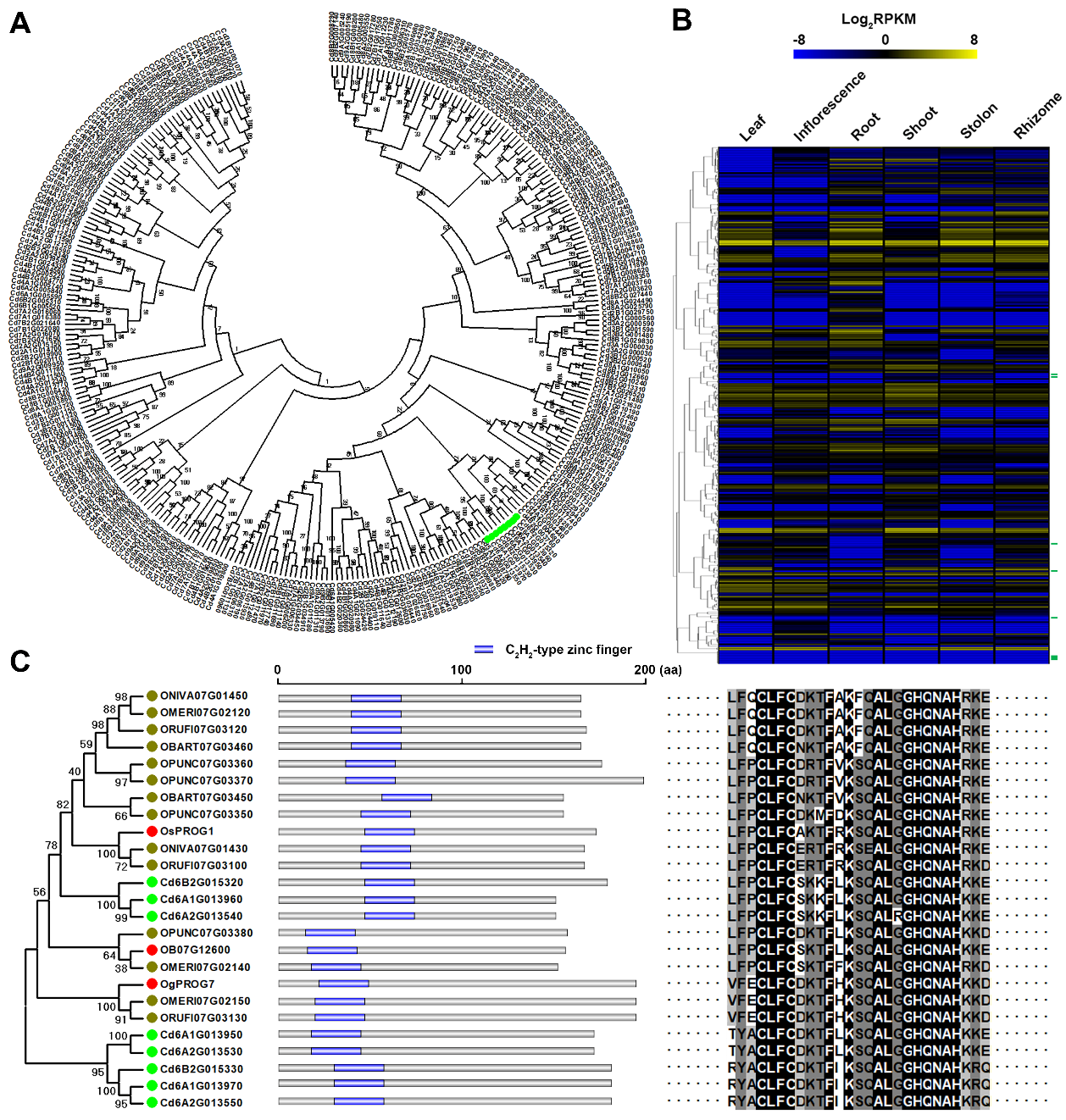
**

**Figure S15. Expansion of *PROG1*-like genes in *C. dactylon***

**A.** Phylogenetic relationships of 305 C_2_H_2_ transcription factors in *C. dactylon* showing the high sequence similarity of eight *PROG1*-like genes (green circles). **B.** Heatmap showing the expression level of the 305 C_2_H_2_ transcription factors in six organs of *C. dactylon*. The eight *PROG1*-like genes were marked in green color. **C**. Phylogenetic relationships and Pfam domain comparison of PROG1-like proteins from *C. dactylon* and eight species of *Oryza* genus with different growth habits. Amino acid sequences of the C_2_H_2_-type zinc finger domain were show in the right panel.

**
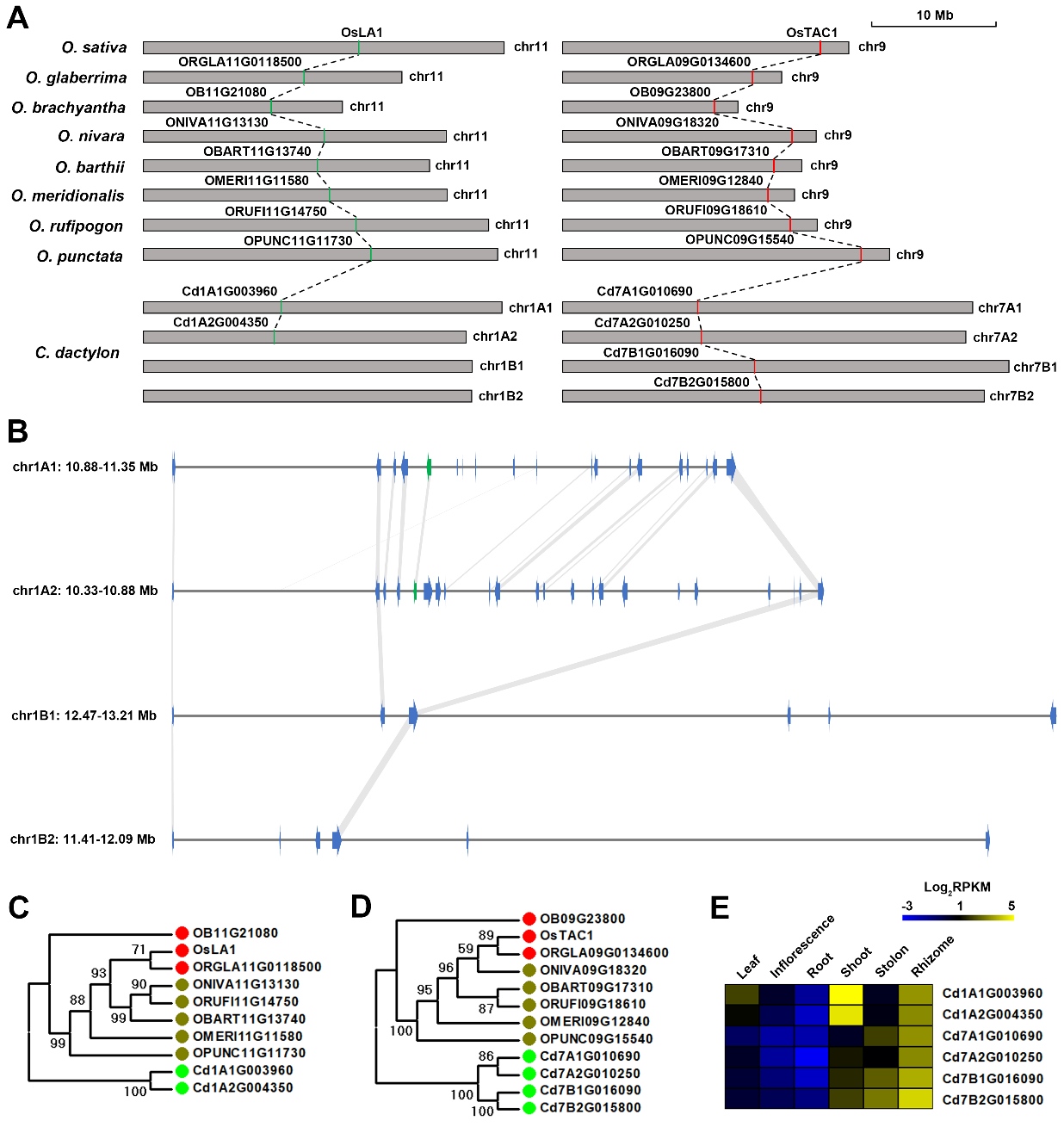
**

**Figure S16. Sequence and expression characteristics of *LAZY1*-like and *TAC1*-like genes in *C. dactylon***

**A.** Chromosome synteny of *LAZY1*-like and *TAC1*-like genes from *C. dactylon* and eight species of *Oryza* genus with different growth habits. **B.** Enlarged chromosomal gene location map showing the loss of *LAZY1*-like gene and other contiguous genes in haplotype B1 and B2 of *C. dactylon*. The *LAZY1*-genes were show in green color. Phylogenetic relationships of **C.** LAZY1-like proteins and **D.** TAC1-like proteins from *C. dactylon* and eight species of *Oryza* genus with different growth habits. **E.** Heatmap showing the expression level of *LAZY1*-like and *TAC1*-like genes in six organs of *C. dactylon*.
